# Macrophage biomimetic nanoparticle-targeted functional extracellular vesicle micro-RNAs revealed via multiomics analysis alleviate sepsis-induced acute lung injury

**DOI:** 10.1186/s12951-024-02597-z

**Published:** 2024-06-23

**Authors:** Guozhen Wang, Xiaoxin Ma, Weichang Huang, Shuanghu Wang, Anni Lou, Jun Wang, Yingfeng Tu, Wanfu Cui, Wangmei Zhou, Wenyong Zhang, Yue Li, Shiyu Geng, Ying Meng, Xu Li

**Affiliations:** 1 Department of Emergency Medicine, Nanfang Hospital, Southern Medical University, Guangzhou 510515, China; 2 State Key Laboratory of Organ Failure Research, Guangdong Provincial Key Laboratory of Viral Hepatitis Research, Southern Medical University, Guangzhou, 510515 Guangdong China; 3 Department of Gastroenterology, Shenzhen People’s Hospital (The Second Clinical Medical College, Jinan University; The First Affiliated Hospital, Southern University of Science and Technology), Shenzhen, 518020 Guangdong China; 4 Central Laboratory, Wenzhou Medical University Lishui Hospital, Lishui People’s Hospital, Lishui, Zhejiang 323000 China; 5 Guangdong Provincial Key Laboratory of Gastroenterology, Department of Gastroenterology, Nanfang Hospital, Southern Medical University, Guangzhou 510515, China; 6 School of Pharmaceutical Science, Guangdong Provincial Key Laboratory of New Drug Screening, Southern Medical University, Guangzhou 510515, China; 7 Department of Intensive Care Unit, General Hospital of Southern Theatre Command, Southern Medical University, Guangzhou 510515, China; 8 Department of Respiratory Diseases, Nanfang Hospital, Southern Medical University, Guangzhou, 510515 China

**Keywords:** Sepsis, Acute lung injury, Extracellular vesicles, Single-cell RNA sequencing, Macrophage membrane nanoparticles

## Abstract

Patients who suffer from sepsis typically experience acute lung injury (ALI). Extracellular vesicles (EVs) contain miRNAs, which are potentially involved in ALI. However, strategies to screen more effective EV-miRNAs as therapeutic targets are yet to be elucidated. In this study, functional EV-miRNAs were identified based on multiomics analysis of single-cell RNA sequencing of targeted organs and serum EV (sEV) miRNA profiles in patients with sepsis. The proportions of neutrophils and macrophages were increased significantly in the lungs of mice receiving sEVs from patients with sepsis compared with healthy controls. Macrophages released more EVs than neutrophils. MiR-125a-5p delivery by sEVs to lung macrophages inhibited Tnfaip3, while miR-221-3p delivery to lung neutrophils inhibited Fos. Macrophage membrane nanoparticles (MM NPs) loaded with an miR-125a-5p inhibitor or miR-221-3p mimic attenuated the response to lipopolysaccharide (LPS)-induced ALI. Transcriptome profiling revealed that EVs derived from LPS-stimulated bone marrow-derived macrophages (BMDMs) induced oxidative stress in neutrophils. Blocking toll-like receptor, CXCR2, or TNFα signaling in neutrophils attenuated the oxidative stress induced by LPS-stimulated BMDM-EVs. This study presents a novel method to screen functional EV-miRNAs and highlights the pivotal role of macrophage-derived EVs in ALI. MM NPs, as delivery systems of key sEV-miRNA mimics or inhibitors, alleviated cellular responses observed in sepsis-induced ALI. This strategy can be used to reduce septic organ damage, particularly lung damage, by targeting EVs.

**Supplementary Information:**

The online version contains supplementary material available at 10.1186/s12951-024-02597-z.

## Introduction

Sepsis is a dysregulated host response to invading pathogens that affects multiple organs due to the occurrence of hemodynamic alterations, pro-inflammatory processes, immune suppression, and endothelial dysfunction [1, 2]. The lung is typically the first organ to become impaired in individuals with sepsis. The clinical term for acute lung injury (ALI), which is a vital prognostic factor that can lead to death in septic patients, is acute respiratory distress syndrome (ARDS) [3]. Symptoms of ALI include dysregulated inflammation, inappropriate accumulation, leukocyte and platelet activation, uncontrolled coagulation pathway activation, and altered permeability of alveolar endothelial and epithelial barriers [4]. ALI involves an exaggerated host-defense immune response caused by an influx of inflammatory cells (e.g., neutrophils and macrophages) into the lung, initiating an inflammatory cycle that perpetuates the accumulation of these cells [5]. Although progress has been made in ARDS treatment, including infection prevention, respiratory support, careful fluid management, nutritional supplementation, and extracorporeal membrane oxygenation [6], the mortality rate for sepsis remains high, with rates of 34.9% in mild cases, 40.3% in moderate cases, and 46.1% in severe cases [7].

Extracellular vesicles (EVs) constitute a heterogeneous population of phospholipid bilayer membrane vesicles secreted by various cell types. They contain nucleic acids (miRNA, long non-coding RNA (lncRNA), cirRNA, and others), proteins, and lipids [8]. The roles of miRNA and the dysregulation of EV-miRNAs in sepsis and sepsis-induced lung injury are diverse [9]. Several miRNAs, such as miR-155, miR-223, miR-146, and miR-27, have shown differential expression levels between sepsis patients and healthy controls. These miRNAs may be useful as biomarkers for sepsis [9]. MiRNAs have been shown to play both protective and harmful roles by regulating the release of inflammatory cytokines [10–15] and aiding in recovery from injury [16]. Various types of lung cells release EVs during sepsis-induced ARDS [17]. Mesenchymal stem cell (MSC) exosomes transmitting miR-23a-3p and miR-182-5p have been utilized to reverse LPS-induced ALI and fibrosis [18]. Neutrophil exosomes carrying miR-30d-5p may induce NF-κB activation, leading to the polarization of M1 macrophages and pyroptosis in the lungs [19]. Bronchoalveolar lavage fluid (BALF) exosomes expressing miR-92a-3p activate NF-κB in alveolar macrophages, resulting in increased inflammation [20]. EV-miRNAs play a significant role in ARDS pathology and are promising therapeutic targets; however, the specific miRNAs that play more effective roles in ALI among the EV-miNRA profiles remain largely unknown. Therefore, new strategies are needed to screen key EV-miRNAs.

Multiomics data, encompassing genomics, epigenomics, transcriptomics, proteomics, metabolomics, and immunomics, offer comprehensive insight into lung disease pathophysiology [21, 22]. Single-cell transcriptomics, the most well-developed of the single-cell omics techniques, is frequently combined with other omics approaches to investigate the correlation between gene expression and phenotypic diversity [23]. In this study, we analyzed the serum EV (sEV) miRNA profiles of patients with sepsis and then investigated the effects of sEVs on the livers and lungs of mice via single-cell RNA (scRNA) sequencing. We identified several functional EV-miRNAs as specific therapeutic targets for sepsis-induced lung injury. MiR-125a-5p was delivered to lung macrophages to inhibit Tnfaip3, while miR-221-3p was delivered to lung neutrophils to inhibit Fos. The generation of DAMPs (damage-associated molecular patterns) and PAMPs (pathogen-associated molecular patterns) results in the release of reactive oxygen species (ROS) by various cells, such as endothelial cells, platelets, neutrophils, and macrophages, during sepsis [24]. This activation further stimulates ROS production by these cells, resulting in a self-perpetuating and expanding ROS activation system [25]. In our research, sepsis patient-derived sEVs promoted ROS production in macrophages, as well as pro-inflammatory cytokine release from macrophages and neutrophils. Macrophages released more EVs than neutrophils. Neutrophil transcriptome profiling revealed that EVs derived from lipopolysaccharide (LPS)-stimulated bone marrow-derived macrophages (BMDMs) induced oxidative stress in neutrophils. Moreover, blocking the toll-like receptor (TLR), CXCR2, or TNFα reduced oxidative stress caused by LPS-BMDM-EVs in the lung.

Recently, biomimetic nanoparticles have gained significant attention owing to their ability to facilitate precise drug delivery with a biomimetic strategy [26]. Packaging an miR-125a-5p inhibitor or miR-221-3p mimic in RAW264.7 membrane nanoparticles has been shown to alleviate sepsis-induced ALI. Thus, we propose a method for screening functional EV micro-RNAs using multiomics-based analysis. Nanovesicles containing an miRNA mimic or inhibitor were constructed by the macrophage membrane (MM) to alleviate sepsis-induced ALI. Several potential inhibitors were found that attenuated macrophage-EV-related lung injury. We provide a novel treatment strategy to reduce sepsis-induced organ damage, especially lung damage, by targeting EVs.

## Results

### Single-cell RNA profiling of liver and lung tissues exposed to EVs derived from the serum of patients with sepsis

**Fig. 1:**
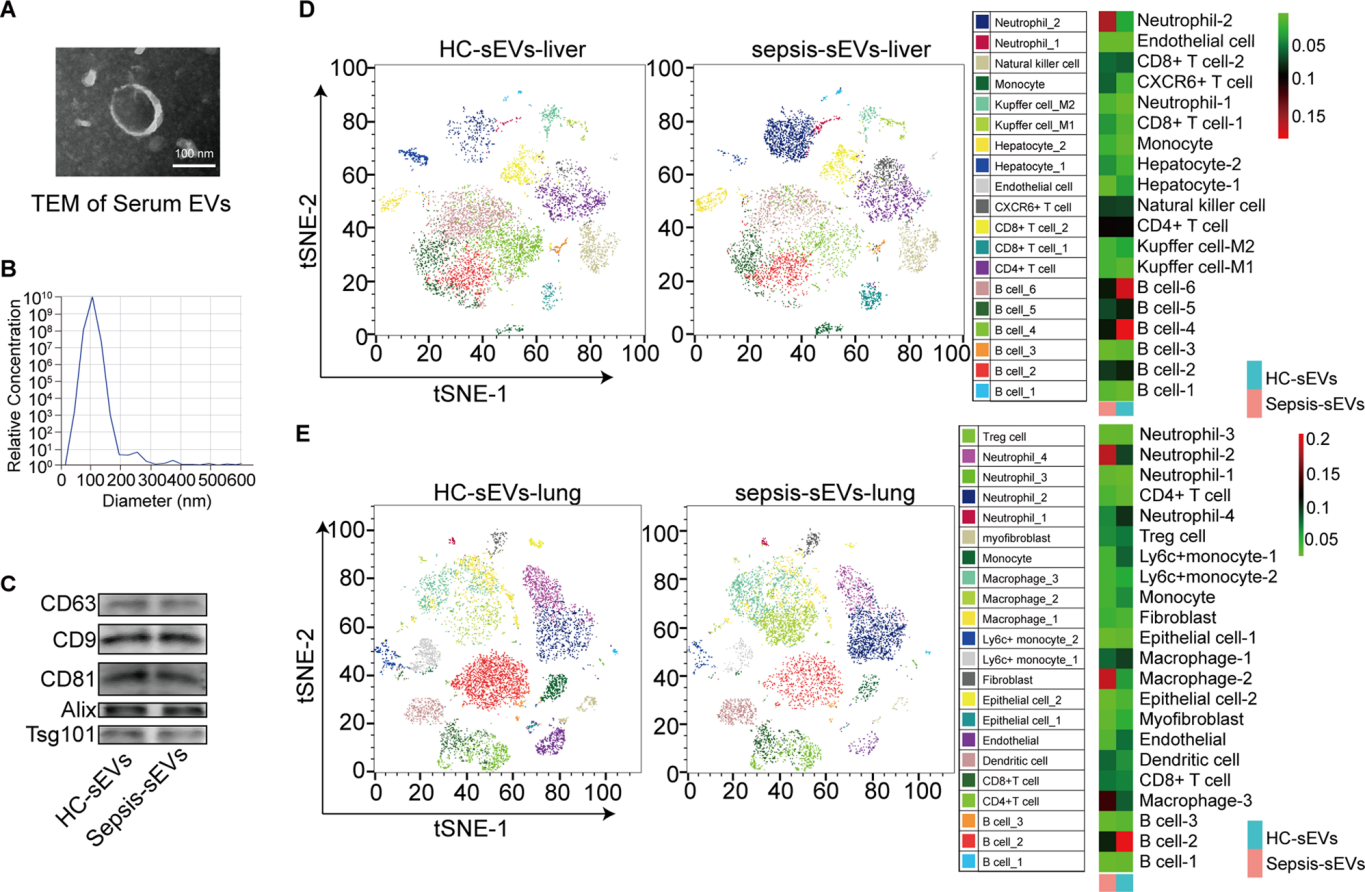
Single-cell RNA profiling of mouse liver and lung tissues exposed to serum extracellular vesicles (sEVs) from healthy individuals or patients with sepsis. (**A**) Transmission electron microscopy of sEVs (∼ 100 nm). (**B**) NTA detection of sEVs. (**C**) Western blotting of sEV biomarkers. (**D**) tSNE plot of the liver uptake of healthy or sepsis sEVs and cell types from scRNA sequencing after liver uptake of healthy or sepsis sEVs. (**E**) tSNE plot of the lung uptake of healthy or sepsis sEVs and cell types from scRNA sequencing after liver uptake of healthy or sepsis sEVs. NTA: Nanoparticle tracking analysis, sEVs: serum extracellular vesicles, HC: Healthy control, and tSNE: t-distributed stochastic neighbor embedding; scale bar: 100 μm

We collected data from patients diagnosed with sepsis using the sequential organ failure assessment (SOFA), which evaluates the functionality of the respiratory, hepatic, cardiovascular, central nervous, and renal systems, as well as the platelet count [27]. Blood culture results suggested a Gram-negative Escherichia coli (E. coli) infection. The patients’ peripheral blood was collected, their serum was separated, and their sEVs were isolated through ultra-centrifugation. Transmission electron microscopy (TEM) showed that the isolated sEVs were approximately 100 nm wide (Fig. 1A). The number of sEVs was determined via nanoparticle tracking analysis (NTA). The sEVs were approximately 100 nm in diameter (Fig. 1B). Biomarkers of sEVs (CD63, CD9, CD81, Alix, and Tsg101) isolated from healthy control subjects and patients with sepsis were detected using western blotting (Fig. 1C).

Immunofluorescence showed that sEVs from the patient with sepsis were taken up in liver and lung tissues (Figure S1). Liver cells were clustered into 19 categories, and lung cells were clustered into 22 cellular compositions, as shown by t-distributed stochastic neighbor embedding (tSNE) (Fig. 1D) via single-cell (SC) transcriptome analysis. The predominant cell populations in the liver were neutrophils, natural killer cells, monocytes, Kupffer cells, hepatocytes, endothelial cells, CXCR6^+^ T cells, CD8^+^ T cells, CD4^+^ T cells, and B cells. The proportion of neutrophils increased, while that of B cells decreased in the livers of mice receiving sEVs from the patient with sepsis versus those receiving healthy control sEVs (Fig. 1D). The primary cell populations in the lung were Treg cells, neutrophils, myofibroblasts, monocytes, macrophages, Ly6C^+^ monocytes, fibroblasts, epithelial cells, endothelial cells, dendritic cells, CD8^+^ T cells, CD4^+^ T cells, and B cells (Fig. 1E). The proportion of neutrophils and macrophages in the lungs of mice receiving sEVs from patients with sepsis was increased, while that of B cells was significantly decreased (Fig. 1E). Immunohistochemical staining revealed that the expression levels of Ly6G (neutrophil marker) in liver and lung tissues and CD68 (macrophage marker) in the lungs of mice that received sEVs from patients with sepsis were significantly higher than those in the lungs of mice that received sEVs from healthy individuals (Figure S2).

### Multiomics-driven analysis of miRNAs in sEVs from patients with sepsis, and scRNA sequencing-based gene expression profiling of mouse liver and lung tissues

**Fig. 2:**
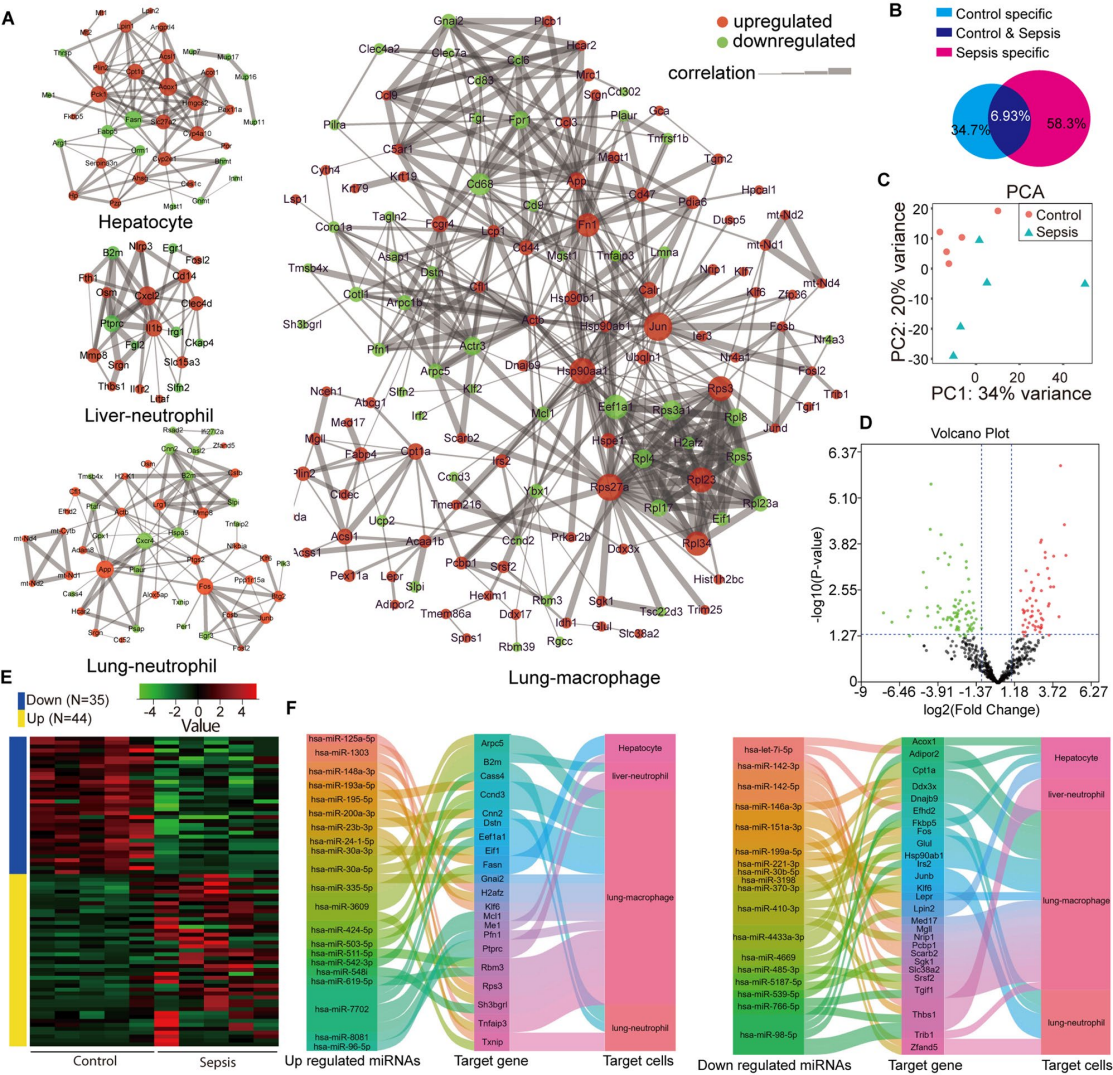
Correlation analysis of miRNAs in serum extracellular vesicles (sEVs) from patients with sepsis, and scRNA gene expression profiles of mouse liver and lung tissues. (**A**) scRNA analysis of gene expression networks in hepatocytes, liver neutrophils, lung neutrophils, and lung macrophages. (**B**) Venn diagram showing the miRNA profiles of healthy and sepsis sEVs (*n* = 5 per group). (**C**) Principal component analysis of the miRNA profiles of healthy and sepsis sEVs (*n* = 5 per group). (**D**) Volcano graph of the miRNA profiles of healthy and sepsis sEVs (*n* = 5 per group). (**E**) Heatmaps of the miRNA profiles of healthy and sepsis sEVs (*n* = 5 per group). (**F**) Predictive analysis of the correlations between miRNAs in sEVs from patients with sepsis and scRNA profiles of hepatocytes, liver neutrophils, lung neutrophils, and lung macrophages

To screen the key sEV-miRNAs in sepsis, we analyzed the differences in the sEV-miRNAs and the gene expression profiles of target cells exposed to sEVs via scRNA sequencing. The potential targets included up- and downregulated mRNAs from hepatocytes, liver neutrophils, lung neutrophils, and lung macrophages, as shown in Fig. 2A. Significant differences in gene expression were observed between the two groups. In hepatocytes, the upregulated genes included Acox1, Slc27a2, Cpt11a, Pck1, Hmgcs2, Cyp4a10, ahsg, Acsl1, Lpin1, Angptl4, and Serpina3n, while the downregulated genes included Fasn, Fabp5, and Orm1. In liver neutrophils, the upregulated genes included CXCL2, NLRP3, IL1β, Clec4d, Srgn, Osm, Fth1, and MMP8, while the downregulated genes included Ptprc, B2m, and Irg1. In lung neutrophils, the upregulated genes included App, Fos, Lrg1, Junb, Btg2, and MMP8, while the downregulated genes included Hspa5, Plaur, Oasl2, Cnn2, and B2m. In lung macrophages, the upregulated genes included Hsp90aa1, Jun, Rps27a, Rps3, App, Fn1, Ccl3, Rpl23, Rpl34, Actb, Cfl1, Fcgr4, and Ccl9, while the downregulated genes included Fpr1, Tnfaip3, Actr3, and Eef1a1 (Fig. 2A).

Next, the results of EV-miRNA sequencing showed that 34.7% of miRNAs were unique to sEVs from healthy individuals, and 58.3% of miRNAs were unique to sEVs from patients with sepsis. The miRNAs in sEVs from both groups accounted for 6.94% (Fig. 2B). Principal component analysis (PCA) revealed significant differences in sEVs between healthy individuals and patients with sepsis (Fig. 2C). Volcano analysis and heatmaps revealed 44 upregulated miRNAs and 35 downregulated miRNAs in sEVs from patients with sepsis relative to those from healthy individuals (Fig. 2D, E). These up- and downregulated miRNAs have the potential to serve as therapeutic targets in sepsis, and the genes that they regulate within target cells are shown in Fig. 2F.

### sEVs delivered miR-125a-5p to lung macrophages and inhibited Tnfaip3, or delivered miR-221-3p to neutrophils and inhibited Fos

**Fig. 3:**
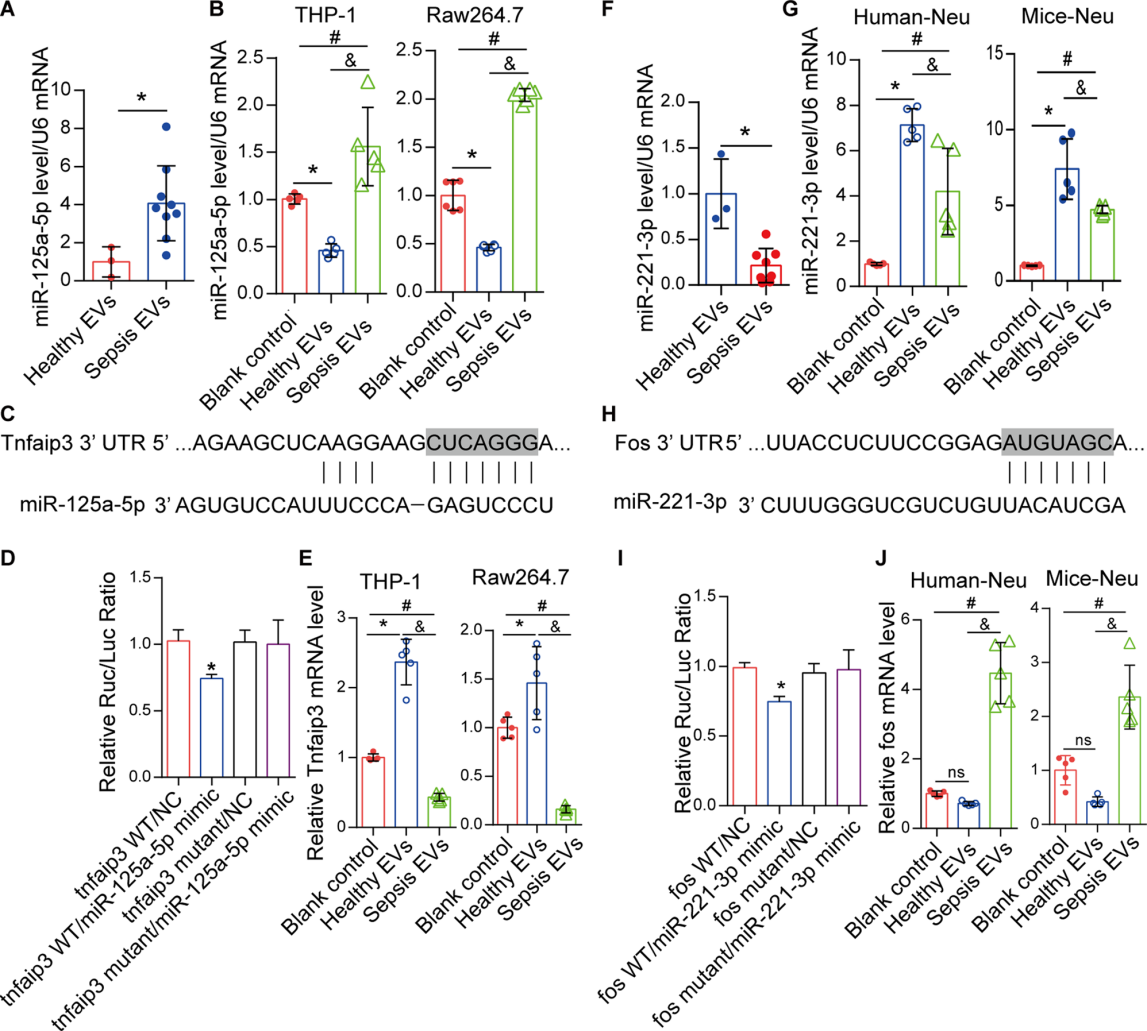
Serum extracellular vesicles (sEVs) deliver miR-125a-5p to lung macrophages and inhibit Tnfaip3, and sEVs deliver miR-221-3p to lung neutrophils and inhibit Fos. (**A**) The level of miR-125a-5p (mean ± SD) in sEVs from healthy individuals (*n* = 3) and patients with sepsis (*n* = 8). *P < 0.05 versus sEVs from healthy individuals. (**B**) THP-1 or Raw264.7 macrophages were stimulated with healthy or sepsis sEVs (*n* = 5 per group), and the intracellular levels of miR-125a-5p (mean ± SD) were detected. *P < 0.05 versus blank control; ^&^*P* < 0.05 versus health-sEVs; and ^#^*P* < 0.05 versus blank control. (**C**) Conserved miR-125a-5p-binding sites in the 3′-UTR of Tnfaip3. (**D**) Dual luciferase activity after 48 h co-transfection of wild-type or mutant 3′-UTRs of mouse Tnfaip3 with miR-125a-5p-NC or lenti-miR-125a-5p-mimic into HEK-293 cells. *P < 0.05 versus Tnfaip3 WT/NC. (**E**) Intracellular levels of Tnfaip3 (mean ± SD) after treatment of THP-1 or Raw264.7 macrophages with healthy or sepsis sEVs (*n* = 5 per group). *P < 0.05 versus blank control; ^&^*P* < 0.05 versus sEVs from healthy individuals; and ^#^*P* < 0.05 versus blank control. (**F**) The level of miR-221-3p (mean ± SD) in sEVs from healthy individuals (*n* = 3) and patients with sepsis (*n* = 8). *P < 0.05 versus sEVs from healthy individuals. (**G**) Primary human or murine neutrophils were exposed to healthy or sepsis sEVs (*n* = 5 per group), and the intracellular levels of miR-221-3p (mean ± SD) were detected. *P < 0.05 versus blank control; ^&^*P* < 0.05 versus health-sEVs; and ^#^: *P* < 0.05 versus blank control. (**H**) Conserved miR-221-3p-binding sites in the 3′-UTR of Fos. (**I**) Dual luciferase activity after 48 h co-transfection of wild-type or mutant 3′-UTRs of mouse Fos with miR-221-3p-NC or lenti-miR-221-3p-mimic into HEK-293 cells. *P < 0.05 versus Fos WT/NC. (**J**) Primary human or murine neutrophils were treated with isolated healthy or sepsis sEVs (*n* = 5 per group), and the intracellular levels of Fos (mean ± SD) were detected. ns: No significance; ^&^*P* < 0.05 versus sEVs from healthy individuals; and ^#^*P* < 0.05 versus blank control. The predicted consequential pairing of the target regions and miRNAs (framed) was based on TargetScan (www.targetscan.org/)

To gain further insight into the sEV-miRNAs that are taken up by target cells, we treated different cell types (macrophages and neutrophils) with sEVs in vitro and examined the changes in miRNA and target gene expression levels (Tnfaip3 and fos, respectively). The levels of miR-125a-5p (Fig. 3A) were significantly higher in sEVs from patients with sepsis than in healthy controls, while those of miR-221-3p (Fig. 3F) were significantly lower in sEVs from patients with sepsis than in healthy controls. Human macrophage Thp-1 and mouse macrophage Raw264.7 cells were selected as recipient cells for miR-125a-5p, while human primary neutrophils and mouse bone marrow neutrophils were selected as target cells for miR-221-3p.

In macrophages, the levels of miR-125a-5p were significantly increased in response to sEVs from patients with sepsis (*P* < 0.01) (Fig. 3B) compared with those from the healthy sEV group. The TargetScan database revealed conserved targets of miR-125a-5p in the 3′-UTR of Tnfaip3 mRNA (Fig. 3C). Overexpression of miR-125a-5p significantly inhibited the luciferase activity of the wild-type Tnfaip3 3′-UTR reporter but not the mutated Tnfaip3 3′-UTR reporter (Fig. 3D). These data indicate that Tnfaip3 is a target of miR-125a-5p. In macrophages, the levels of Tnfaip3 decreased significantly in response to sEVs from patients with sepsis (*P* < 0.01) (Fig. 3E) compared with those from the healthy sEV group.

In neutrophils, the levels of miR-221-3p were significantly higher in the healthy sEV group (*P* < 0.01) (Fig. 3G) than in the sepsis sEV group. The TargetScan database revealed conserved targets of miR-221-3p in the 3′-UTR of Fos mRNA (Fig. 3H). Overexpression of miR-221-3p significantly inhibited the luciferase activity of the wild-type Fos 3′-UTR reporter but not the mutated Fos 3′-UTR reporter (Fig. 3I). These data indicate that Fos is a target of miR-221-3p. The levels of Fos in response to sEVs from patients with sepsis were significantly higher (*P* < 0.01) (Fig. 3J) than those in response to healthy sEVs.

### Raw264.7 MM NPs containing miR-125a-5p inhibitor or miR-221-3p mimic reduced LPS-induced ALI

**Fig. 4:**
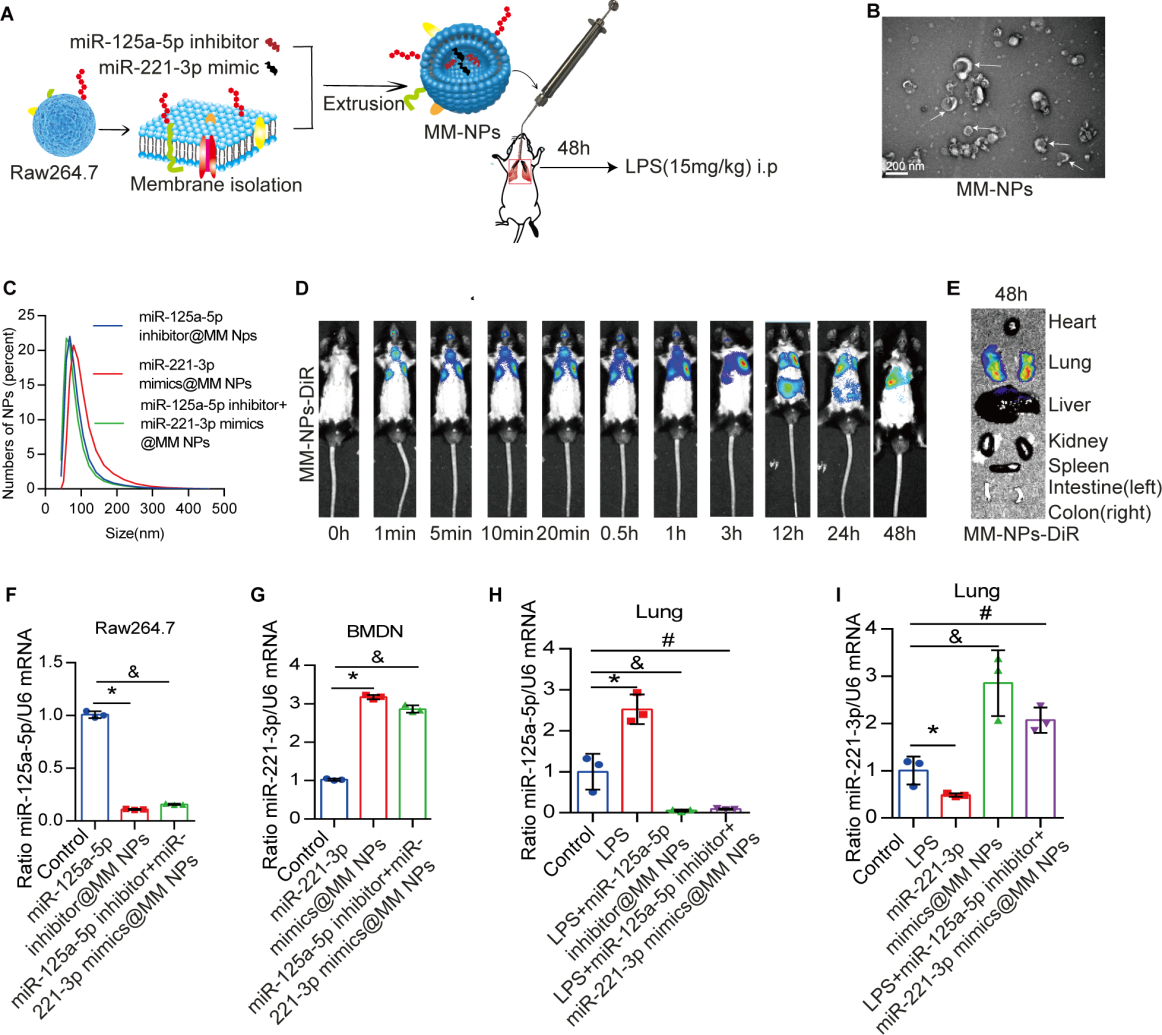
Raw264.7 membrane-packaged miR-125a-5p inhibitor or miR-221-3p mimic. (**A**) Protocol for packaging miR-125a-5p inhibitor or miR-221-3p mimic in Raw264.7 membrane nanoparticles. (**B**) TEM of MM NPs after packaging with miR-125a-5p inhibitor and miR-221-3p mimic. (**C**) Diameter of miR-125a-5p inhibitor@MM NPs, miR-221-3p mimic@MM NPs, and miR-125a-5p inhibitor + miR-221-3p mimic@MM NPs, as detected using DLS (*n* = 3 per group). (**D**, **E**) In vivo imaging of Dir-stained MM showed that NPs were primarily concentrated in the lungs. (**F**) Intracellular miR-125a-5p (mean ± SD, *n* = 3 per group) in Raw264.7 cells treated with control MM NPs, miR-125a-5p inhibitor@MM NPs, miR-221-3p mimic@MM NPs, or miR-125a-5p inhibitor + miR-221-3p mimic@MM NPs. *P < 0.05 versus control; ^&^*P* < 0.05 versus control. (**G**) Intracellular miR-1221-3p (mean ± SD, *n* = 3 per group) in BMDNs exposed to control MM NPs, miR-125a-5p inhibitor@MM NPs, miR-221-3p mimic@MM NPs, or miR-125a-5p inhibitor + miR-221-3p mimic@MM NPs. *P < 0.05 versus control; ^&^*P* < 0.05 versus control. (**H**, **I**) miR-125a-5p inhibitor@MM NPs, miR-221-3p mimic@MM NPs, or miR-125a-5p inhibitor + miR-221-3p mimic@MM NPs was administered intratracheally into wild-type C57BL/6 mice for 48 h before intraperitoneal injection of LPS. miR-125a-5p and miR-221-3p (mean ± SD, *n* = 3 per group) were detected in the lung using RT-PCR. *P < 0.05 versus control; ^&^*P* < 0.05 versus control; and ^#^*P* < 0.05 versus control

Recently, biomimetic nanoparticles have gained attention for their ability to precisely deliver drugs [26]. Based on a mixture of miR-125a-5p inhibitors (or miR-221-3p mimics) and MM, which was extruded through a 200-nm membrane, three types of MM NPs were produced: miR-125a-5p inhibitor@MM NPs, miR-221-3p mimic@MM NPs, and miR-125a-5p inhibitor + miR-221-3p mimic@MM NPs (Fig. 4A). The MM NPs exhibited a double-membrane structure with a diameter of ∼ 140 nm, as revealed by TEM and DLS (Fig. 4B, C). The loading efficiency of miR-125a-5p inhibitors (or miR-221-3p mimics) was 9.464 ± 0.8705% ( or 8.135 ± 1.276%), as determined by the fluorescence intensity of Alexa Flour 488-labeled miR-125a-5p inhibitors (or miR-221-3p mimics) in MM NPs (Figure S3A). Meanwhile, the loading efficiency of miR-125a-5p inhibitors + miR-221-3p mimics in MM NPs was 6.118 ± 0.2671% (Figure S3A). In vivo imaging revealed that the MM NPs were primarily concentrated in the lungs at 1 min to 48 h after tracheal administration (Fig. 4D–E). MM NPs were labeled with Fluorescein Isothiocyanate (FITC), and the percentage of macrophages absorbing FITC-MM NPs was determined using flow cytometry, revealing that 46.20 ± 1.77% of FITC labeled MM NPs were targeted to lung macrophages after tracheal administration (Figure S3B). MiR-125a-5p inhibitor@MM NPs significantly inhibited the miR-125a-5p in Raw264.7 cells, whereas miR-221-3p mimic@MM NPs significantly increased the miR-221-3p in BMDNs. As expected, the miR-125a-5p inhibitor + miR-221-3p mimic@MM NPs inhibited miR-125a-5p in Raw264.7 cells and increased miR-221-3p in BMDNs (Fig. 4F–G).

LPS significantly increased miR-125a-5p in the lungs and decreased miR-221-3p compared with the control group (Fig. 4H, I). Administration of miR-125a-5p inhibitor@MM NPs before LPS treatment significantly reduced the level of miR-125a-5p in the lungs, and pre-treatment with miR-221-3p mimic@MM NPs increased the level of miR-221-3p. Administration of miR-125a-5p inhibitor + miR-221-3p mimic@MM NPs before LPS treatment reduced the level of miR-125a-5p in the lungs and increased the level of miR-221-3p (Fig. 4H–I).

**Fig. 5:**
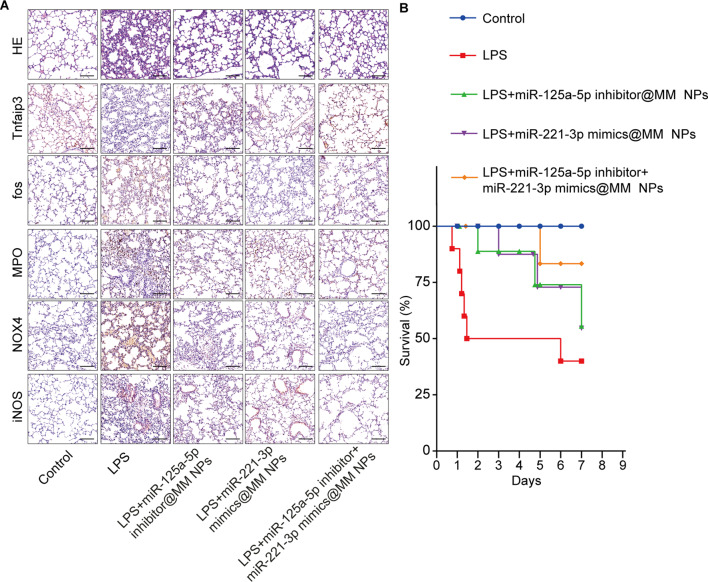
Raw264.7 membrane-packaged miR-125a-5p inhibitor or miR-221-3p mimic reduced LPS-induced ALI. (**A**) Lung tissue was analyzed via H&E staining, and the expression levels of Tnfaip3 and Fos were analyzed using IHC; scale bar: 100 μm. (**B**) Survival analysis of mice treated with miR-125a-5p@MM NP, miR-221-3p mimic@MM NPs, and miR-125a-5p inhibitor + miR-221-3p mimic@MM NPs exposed to LPS (15 mg/kg). MM NPs: Macrophage membrane nanoparticles, H&E: Hematoxylin-eosin, and IHC: Immunohistochemistry

In mice that received LPS alone, lung histology showed that the alveolar space was widened and that the interstitial blood vessels exhibited obvious congestion and infiltration of inflammatory cells. Additionally, the expression levels of Tnfaip3 decreased, whereas those of Fos increased in the LPS group (Fig. 5A) compared with those in the control group. Pre-treatment with miR-125a-5p inhibitor@MM NPs or miR-125a-5p inhibitor + miR-221-3p mimic@MM NPs increased the expression of Tnfaip3, whereas pre-treatment with miR-221-3p mimic@MM NPs or miR-125a-5p inhibitor + miR-221-3p mimic@MM NPs decreased the expression of Fos compared with the LPS group (Fig. 5A).

The levels of MPO, NOX4, and inducible nitric oxide synthase (iNOS) in the LPS group were significantly increased compared with those in the control group. Pre-treatment with miR-125a-5p inhibitor@MM NPs or miR-221-3p mimic@MM NPs reduced LPS-induced ALI and decreased the expression of MPO, NOX4, and iNOS (Fig. 5A). Moreover, pre-treatment with miR-125a-5p inhibitor + miR-221-3p mimic@MM NPs attenuated ALI, and substantial expression of MPO, NOX4, and iNOS was observed compared with that in pre-treatment with miR-125a-5p inhibitor@MM NPs or miR-221-3p mimic@MM NPs (Fig. 5A).

Survival analysis revealed that pre-treatment with miR-125a-5p@MM NPs or miR-221-3p mimic@MM NPs significantly improved the survival of mice compared with non-pretreated mice. Moreover, the survival time of mice pre-treated with miR-125a-5p inhibitor + miR-221-3p mimic@MM NPs was better than that of mice that received miR-125a-5p@MM NPs or miR-221-3p mimic@MM NPs (Fig. 5B).

### Multiomics analysis of proteins in sEVs from patients with sepsis, and signaling pathway enrichment analysis of mouse liver and lung tissues

In addition to miRNAs, EVs contain proteins that contribute to sepsis-induced ALI. Owing to the technical limitations of conducting single cell (SC) mass spectrometry analysis, we adopted a strategy based on SC sequencing to analyze signaling pathway activation. Correlation analysis was conducted between activated signaling pathways and those associated with EV-associated proteins.

**Fig. 6:**
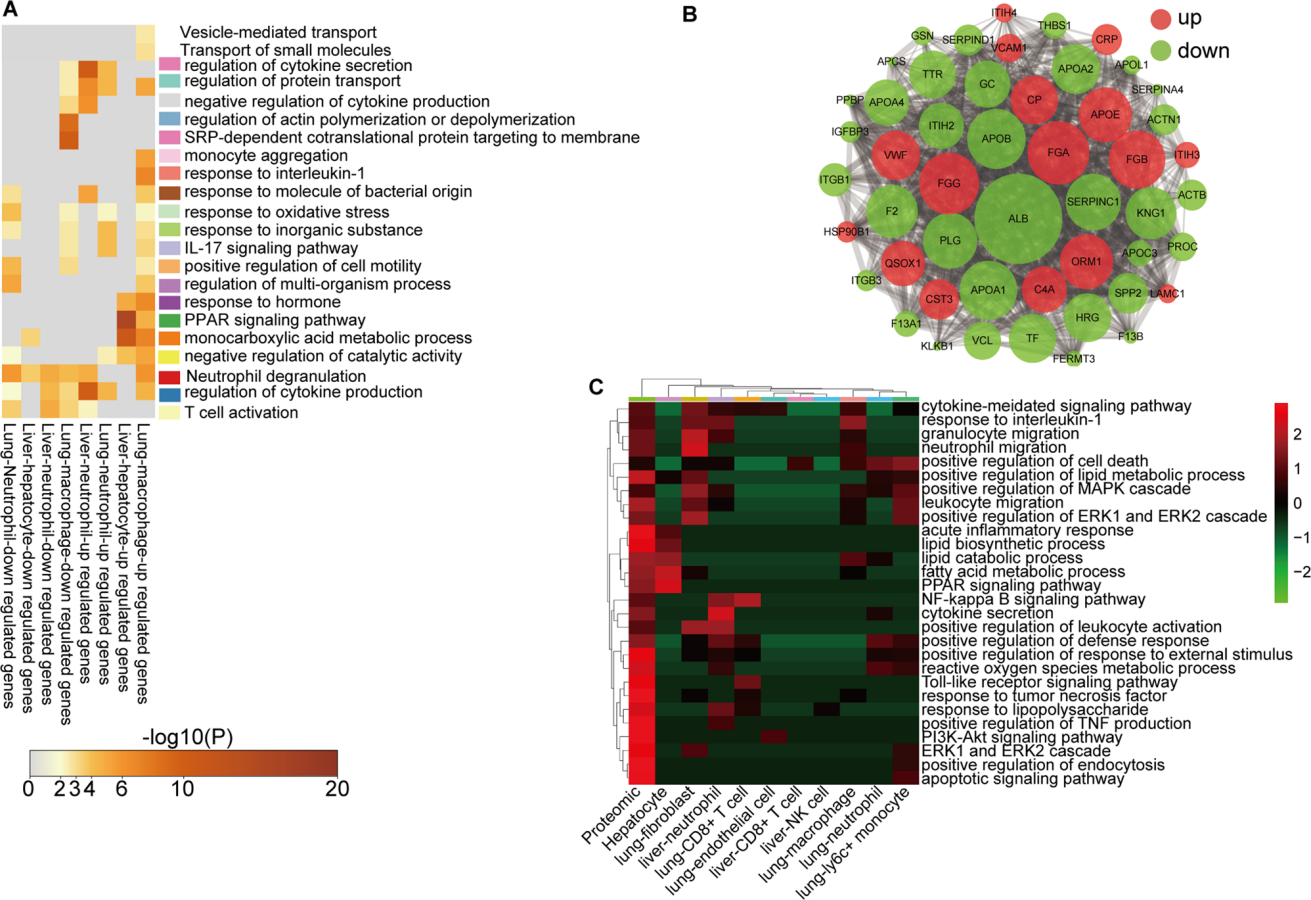
Multiomics analysis of proteins in sEVs from patients with sepsis, and liver and lung signaling pathway activation profiling. (**A**) Gene Ontology (GO) analysis of differentially expressed genes (DEGs) in hepatocytes, liver neutrophils, lung neutrophils, and lung macrophages. (**B**) Analysis of the protein networks in sEVs from healthy individuals and patients with sepsis. (**C**) Combined GO analysis of the sEV proteome and DEGs in target cells

The Gene Ontology (GO) analysis of the genes that were upregulated in the sepsis sEV group in several cell types (lung macrophages, lung neutrophils, liver neutrophils, and hepatocytes) compared with that of the healthy control sEV group is shown in Fig. 6A. Sepsis sEVs may promote ROS production in macrophages, pro-inflammatory cytokine release from macrophages and neutrophils, and PPAR signaling in hepatocytes.

We treated Raw264.7 macrophages with healthy or sepsis sEVs to assess pro-inflammatory cytokine expression using RT-PCR analysis. The results showed that the levels of Tnfα, IL-1β, CXCL2, CXCL10, CCL2, and CCL3 increased significantly in response to sepsis sEVs compared with healthy sEVs (Figure S4A). Regarding neutrophils, the levels of Tnfα, IL-6, IL-1β, CXCL1, CXCL2, CCL5, and CXCL10 increased significantly in response to sepsis sEVs compared with those from the control group and healthy individuals (Figure S4B). Moreover, the ROS levels increased significantly in response to sepsis sEVs compared with healthy sEVs (Figure S4C). The results of mass spectrometry analysis of proteins within sEVs demonstrated significant differences in the protein compositions of sEVs from healthy individuals and patients with sepsis (Figure S5A). Volcano plot analysis and heatmaps revealed that 79 proteins were increased and 97 proteins were decreased in response to sEVs from patients with sepsis versus sEVs from healthy individuals (Figure S5B, C). GO analysis and enriched signaling pathways are shown in Figure S5D–E. Protein-protein interaction analysis revealed that upregulated proteins included FGG, FGA, CP, FGB, APOE, ORM1, and CRP, while downregulated proteins included ALB, APOB, Serpinc1, APOA1, PLG, F2, and KNG1 (Fig. 6B).

The combined GO analysis of the sEV proteome and differentially expressed genes (DEGs) in target cells is shown in Fig. 6C. We propose that some proteins in sEVs from patients with sepsis could be involved in the biological functions of diverse target cells, which may include the following: granulocyte migration, neutrophil migration, positive regulation of leukocyte activation, and positive regulation of the ERK1 and ERK2 cascade in lung fibroblasts; fatty acid metabolic process, PPAR signaling pathway, and lipid catabolic process in hepatocytes; cytokine secretion, NF-κB signaling pathway, positive regulation of leukocyte activation, and response to LPS in liver neutrophils; and NF-κB signaling pathway and TLR signaling pathway in lung-CD8^+^ T cells. Furthermore, the list includes PI3K-Akt signaling pathway in lung endothelial cells; positive regulation of cell death in liver-CD8^+^ T cells; response to interleukin-1 in lung macrophages; positive regulation of defense response and ROS metabolic process in lung neutrophils; and positive regulation of MAPK cascade, leukocyte migration, and positive regulation of the ERK1 and ERK2 cascade in lung Ly6c^+^ monocytes (Fig. 6C). These results suggest that sEV proteins also participate in the regulation of target cell function.

In addition to miRNAs and proteins, sEVs isolated from patients with sepsis drive pro-inflammatory responses similar to those induced by LPS alone. However, we detected no significant difference in LPS levels between sEVs from the healthy controls and patients with sepsis (Figure S6).

### Macrophages released more EVs than neutrophils

**Fig. 7:**
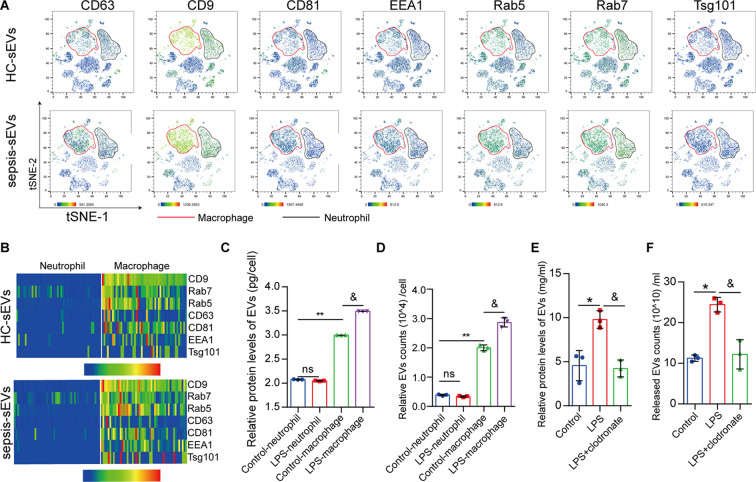
Macrophages release more EVs than neutrophils. (**A**) tSNE plot of EV markers (CD63, CD9, CD81, and Tsg101) and endosomal markers (EEA1, Rab5, Rab7) in the lungs of mice exposed to healthy and sepsis sEVs. (**B**) Heatmap of EV markers (CD63, CD9, CD81, and Tsg101) and endosomal markers (EEA1, Rab5, and Rab7) in the lung neutrophils and macrophages of mice exposed to healthy and sepsis sEVs. (**C**, **D**) Relative protein levels (mean ± SD, *n* = 3 per group) and numbers of EVs (mean ± SD, *n* = 3 per group) released by human peripheral neutrophils and macrophages with or without LPS treatment. ns: No significance; **P < 0.01 versus control neutrophil; and ^&^*P* < 0.05 versus control macrophage. (**E**, **F**) Relative protein levels (mean ± SD, *n* = 3 per group) and numbers of EVs (mean ± SD, *n* = 3 per group) released from the lung alveolar lavage fluid in the control, LPS, and LPS + clodronate (macrophage scavenger) groups. *P < 0.01 versus control; ^&^*P* < 0.05 versus LPS. HC: Healthy control

During sepsis, lung resident cells and recruited immune cells can release EVs, which may represent therapeutic targets for sepsis. As shown in Fig. 1F, the proportion of neutrophils and macrophages in the lungs of mice that received sEVs from patients with sepsis was increased compared with that in the healthy control sEV group. scRNA analysis revealed that the EV markers CD63, CD9, CD81, and Tsg101 were mainly expressed in lung macrophages, while the endosomal markers EEA1, Rab5, and Rab7 were highly expressed in macrophages (Fig. 7A). Heatmaps revealed that the expression levels of CD63, CD9, CD81, Tsg101, EEA1, Rab5, and Rab7 in lung macrophages were significantly higher than those in neutrophils in the healthy control sEV group and sEVs from patients with sepsis (Fig. 7B).

LPS treatment increased the number of EVs released by macrophages but did not significantly affect the number of EVs released by neutrophils (Fig. 7C–D). Moreover, macrophages released many more EVs than neutrophils with or without LPS stimulation (Fig. 7C–D).

Clodronate to clear macrophages from the lungs (Figure S7A). LPS treatment significantly increased the protein concentration of lung alveolar lavage fluid-EVs released in the lung relative to the control group, while pre-treatment with clodronate significantly decreased the protein levels of lung alveolar lavage fluid-EVs compared with the LPS group (Fig. 7E). The number of EVs detected using NTA showed the same pattern (Fig. 7F). The findings suggest that EVs in the lungs are predominantly of macrophage origin, implicating them in the development of sepsis-induced ALI.

### Inhibiting TLR, CXCR2, or Tnfα reduced the lung oxidative stress caused by LPS-stimulated macrophage EVs

**Fig. 8:**
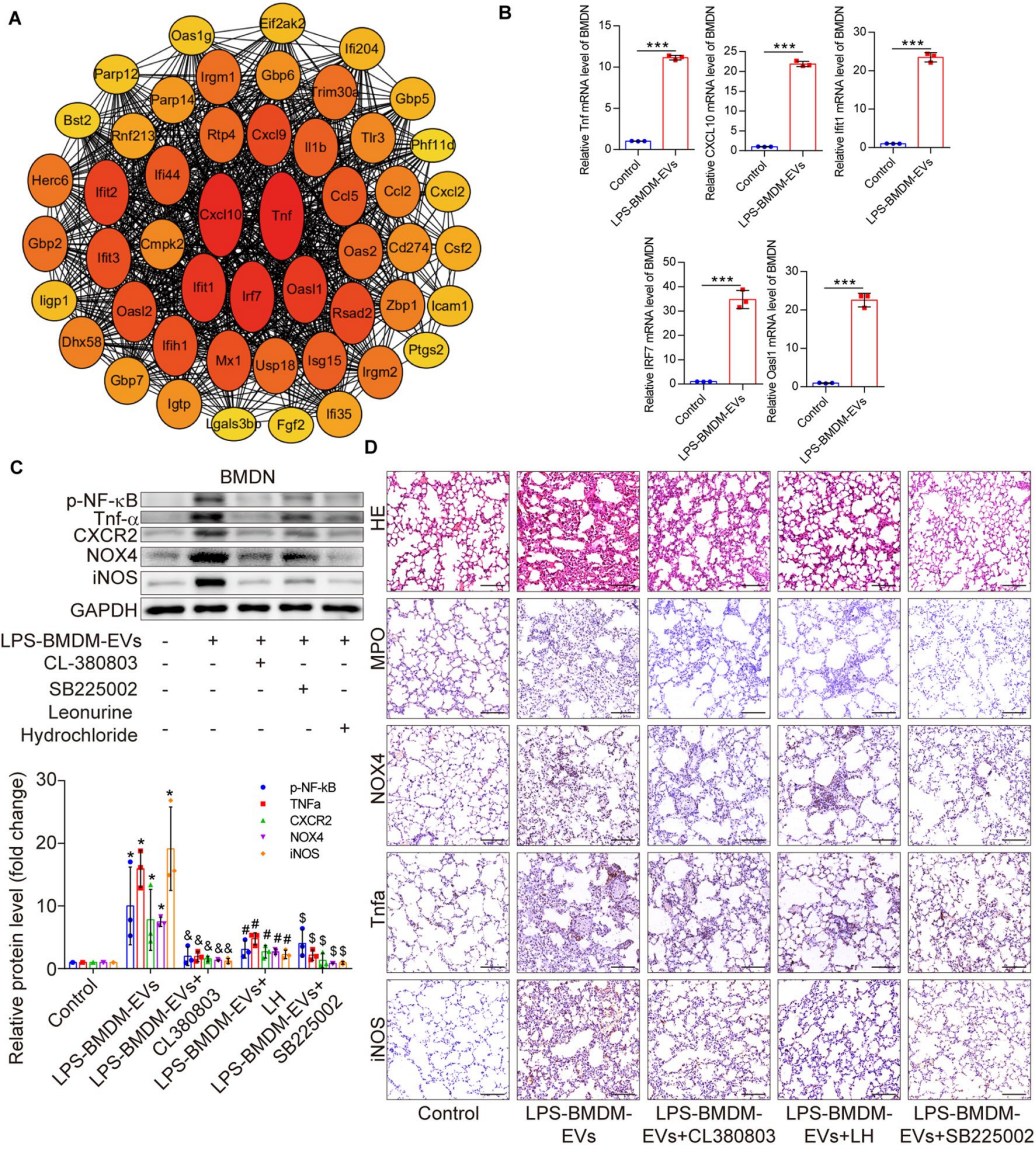
Inhibition of TLR, CXCR2, or Tnfα reduces the oxidative stress in the lung caused by EVs derived from LPS-stimulated macrophages. (**A**) The network of upregulated genes in neutrophils treated with LPS-BMDM-EVs relative to the control group. (**B**) The mRNA levels (mean ± SD, *n* = 3 per group) of TNF, CXCL10, Ifit1, IRF7, and Oasl1 in BMDNs from the control and LPS-BMDM-EV groups. ***P < 0.001 versus control. (**C**) The expression levels of NF-κB, p-NF-κB, Tnfα, CXCR2, NOX4, and iNOS (mean ± SD, *n* = 3 per group) of BMDNs stimulated with LPS-BMDM-EVs and then treated with CL-380,803, SB225002, or leonurine hydrochloride (LH). *P < 0.05 versus control; ^&^*P* < 0.05 versus LPS-BMDM-EVs; ^#^*P* < 0.05 versus LPS-BMDM-EVs; and ^$^*P* < 0.05 versus LPS-BMDM-EVs. (**D**) H&E staining and expression levels of MPO, NOX4, Tnfα, and iNOS in the lungs of the BMDM-EV treatment group after administration of CL-380,803, SB225002, or LH by tail vein injection (*n* = 6 per group); scale bar: 100 μm. BMDM: Bone marrow-derived macrophages, BMDN: Bone marrow-derived neutrophils, and H&E: hematoxylin and eosin

As macrophages released more EVs than neutrophils and were recruited into the lung during sepsis, we speculated that EVs derived from LPS-stimulated macrophages might affect the biology of neutrophils. The results of transcriptome sequencing revealed that LPS-stimulated bone marrow-derived macrophage EVs (LPS-BMDM-EVs) upregulated 1,034 genes and downregulated 786 genes in bone marrow-derived neutrophils (BMDNs) when compared with the control group (Figure S8A, C). PCA revealed significant differences between the control and LPS-BMDM-EV groups (Figure S8B). The biological processes, cellular components, and molecular functions of the upregulated genes in the LPS-BMDM-EV group are shown in Figure S8D-F. The pathways affected by upregulated genes are shown in Figure S8G. Analysis of the interaction network between upregulated genes and the LPS-BMDM-EVs included Tnfα, CXCL10, Irf7, Ifit1, and Oasl1 (Fig. 8A).

The levels of CXCL10, Tnfα, Ifit1, Irf7, and Oasl1 were significantly higher in the LPS-BMDM-EV group than in the control group, as measured using qPCR (Fig. 8B). We treated BMDNs with inhibitors of TLR signaling (Figure S9A), CXCR signaling (Figure S9B), and Tnfα signaling (Figure S9C), after exposing the cells to LPS-BMDM-EVs.

The addition of leonurine hydrochloride (LH; a TLR antagonist), SB225002 (a CXCR2 antagonist), and CL-380,803 (a Tnfα antagonist) reduced the ROS level in BMDNs compared with the untreated control cells (LPS-BMDM-EV group). The levels of p-NF-κB, Tnfα, CXCR2, NOX4, and iNOS increased significantly in the treatment group compared with the control group. LH, SB225002, and CL-380,803 inhibited the expression of p-NF-κB, Tnfα, CXCR2, NOX4, and iNOS compared with the LPS-BMDM-EV group (Fig. 8C). Hematoxylin and eosin staining of the lung samples revealed interstitial edema accompanied by inflammatory cell infiltration in the untreated LPS-BMDM-EV group compared with the treated group. The expression levels of MPO, NOX4, Tnfα, and iNOS increased significantly following exposure to LPS-BMDM-EVs, whereas treatment with CL-380,803, SB225002, and LH reduced lung inflammation and the expression levels of MPO, NOX4, Tnfα, and iNOS (Fig. 8D).

## Discussion

As comprehensive studies of EVs from patients with sepsis are rare, we used sequencing technology and mass spectrometry to profile the miRNAs and proteins within sEVs. We analyzed genetic changes in lung and liver cells in response to sEVs via scRNA sequencing. We established a key EVs-miRNA screening method using correlation prediction analysis of the SC transcriptome and EV-miRNA target gene profiles. We predicted a correlation between EV-miRNAs and the gene expression profile of specific target cells. Our results provide evidence that EV-miRNAs play multiple roles in different cell types. However, this study also has limitations. First, the influence of EVs on the mRNA expression profile of specific cells is diverse because EVs contain multiple miRNAs; therefore, the effects of delivering specific miRNAs to target cells remain to be elucidated. Second, changes in the mRNA expression profile in target cells are affected by various factors, including EV proteins and other elements within the target cells. Our mass spectrometry data indicated that sEV proteins affect biological functions in target cells; however, enrichment analysis showed that the sEV proteins are more involved in regulating protein binding, which may be related to the uptake of sEVs by target cells. Finally, the correspondence between sEV-miRNAs and target genes of specific cells obtained by this prediction method remains to be confirmed experimentally.

We confirmed that EVs deliver miR-125a-5p and miR-221-3p to macrophages and neutrophils, respectively. MiR-146a, which represses the expression of IRAK-1 and TRAF-6 and suppresses inflammatory mediators, is the best-studied miRNA participating in ALI [10]. Recently, Alexander et al. [28] reportedobserved that dendritic cell-derived exosomes contain miR-146a and miR-155, which are transferred between immune cells in vivo. Their study also revealed that exosomes containing miR-146a diminish the expression of inflammatory genes, while those containing miR-155 promote this process. Further research is necessary to better understand the effects of EV-mediated miRNA transfer in ALI and the underlying mechanisms.

In bacterial pneumonia, macrophages release a significant amount of apoptotic bodies (ABs) enriched in miR-221 and miR-222 [29]. Marlene Reithmair reported that three extracellular miRNAs (miR-30a-5p in exosomes, miR-125b-5p, and miR-193a-5p in serum) predict survival with high confidence [30]. Our miRNA sequencing data reveal that the levels of miR-146a-3p and miR-125a-5p are lower and higher, respectively, in sEVs from patients with sepsis than in those from healthy individuals. In this study, miRNAs were screened based on the combined analysis of SC and miRNA sequencing data; miRNA identified in this manner may offer more meaningful therapeutic targets.

Furthermore, according to Moon et al., alveolar macrophages (AMs) are the primary sources of the micro vesicles (MVs) detected in bronchoalveolar lavage fluid (BALF) in mice without exposure to noxious stimuli [31]. Bacterial infection frequently induces extensive pro-inflammatory responses to elicit bactericidal effects. Presumably, after inhaling bacteria or LPS, AMs are the primary responders and likely release EVs into BALF. AM-derived apoptotic bodies can influence epithelial cell function, possibly enhancing epithelial proliferation, suggesting two-way communication between AMs and epithelial cells, rather than unidirectional signaling [29]. EVs carrying macrophage markers are highly upregulated after exposure to LPS or *P. pneumoniae*. Additionally, EVs carrying endothelial and polymorphonuclear neutrophil (PMN) markers are slightly increased, while those carrying the macrophage marker CD68 are substantially increased after exposure to LPS or *P. pneumoniae* [32]. We also found that macrophages are the primary source of EVs in LPS-induced ALI. However, instead of isolating EVs to detect specific cell-type markers, we evaluated EVs via SC transcriptome sequencing to determine their potential for releasing EV markers, which is more aligned with in vivo conditions. We observed no significant changes in the markers of EVs released by lung epithelial cells, which may be related to the degree of epithelial cell death in sepsis-induced ALI.

We also constructed a drug-carrying system for miRNA mimics and inhibitors using MM NPs. MiRNAs play a crucial role in the pathophysiology of ALI. The transfer of EV-miRNAs to various types of lung cells contributes to the disease process [33]. Therefore, EV-mediated miRNAs are considered promising targets for novel cell-specific therapeutic approaches. Employing EVs as a means of transporting exogenous nucleotides has several benefits compared to other methods of delivery mediated by nanoparticles [34]. Microvesicles (MVs) can be obtained from the host’s blood [34]. Zhang et al. delivered a miR-223/142 mimic in vivo using MVs [13]. In this study, the MM NPs’ macrophage-specific delivery mechanism has the potential to be more effective and cause fewer off-target effects when using miRNA molecules as therapeutics. For instance, Ohno et al. conducted an early proof-of-concept study showing that exosomes from human embryonic kidney cells could efficiently convey exogenous therapeutic let-7a in EGFR-expressing xenograft breast cancer tissues from RAG2^(–/–)^ mice, leading to tumor regression [35]. Our method of collecting EVs has several advantages over previous methods that alter the expression of protein or RNA in donor cells and then collect the released EVs. In our method, the donor cells are not modified, and there is no need to culture large numbers of cells. Moreover, the type and quantity of the packaged cargos are more flexible and have higher purity, and interference between cargos can be avoided, as well as the protocol being rapid and straightforward to perform. However, there are also limitations, including the unclear efficiency of EV uptake by different cell types.

Further study is necessary to determine how to deliver miRNA-based therapeutics to specific organs and target cell types, including alveolar ECs and alveolar macrophages, which play a critical role in lung diseases. In addition, it is important to identify the long-term effects of miRNA mimics and inhibitors, since these compounds can persist in tissues for several months [36]. Furthermore, as one miRNA can affect several molecular pathways, unintended effects are possible. Future research should focus on clarifying the roles of EV-miRNAs in the development of lung diseases and providing relevant information to enhance the development of new diagnostics and therapeutics. The mechanisms by which EV-miRNAs are taken up by recipient cells, including their receptors, remain unclear and warrant further investigation.

The temporal pattern of EV release during early ALI was analyzed by Soni et al., and they discovered that alveolar macrophages are the primary source of MVs, which play a significant role in instigating the inflammatory response, in part by transferring TNF to the target cells [37]. The binding of LPS to the TLR initiates a complex sequence of events that elevates the expression of specific pro-inflammatory genes through NF-κB, including Tnfα and iNOS [38]. Agents capable of modulating systemic inflammation may represent potential treatments for severe sepsis. We also found that LPS-macrophage EVs primarily activate the TLR, CXCR, and TNF signaling pathways in neutrophils, while inhibiting these pathways reduces lung inflammation and oxidative stress caused by LPS-BMDM-EVs.

Exosomes have been found to affect various types of immune cells during sepsis. They activate NF-κB and promote cytokine production, including IL-1β, IL-6, IL-12, and TNF-α, in macrophages [39–41]. In sepsis-induced ALI and ARDS, both BALF and circulating EVs show an increase in quantity [42, 43]. However, the role of alveolar macrophage-derived EVs in ALI is also complex because they not only participate in ALI as pro-inflammatory factors, but also attenuate lung injury during ARDS. This dual function may be related to phenotypic changes in macrophages. In the early stages of ALI, the majority of resident macrophages immediately convert to the M1 phenotype, serving as the first line of defense against pathogens and lung tissue injury, while M2 macrophages, which convert in the late stage of sepsis, limit the levels of pro-inflammatory factors, produce anti-inflammatory cytokines, and phagocytose apoptotic neutrophils. Our results showed that sepsis patient-derived sEVs promoted ROS production in macrophages, as well as pro-inflammatory cytokine release from macrophages and neutrophils. In contrast to the previous literature, the cellular origin of sepsis patient-derived sEVs is more complex than that of BALF-derived EVs, and the pro-inflammatory effects we observed from sepsis patient-derived sEVs may be related to the different phases of sepsis.

In this study, we focused on the transportation of EVs from lung macrophages to lung neutrophils, although EV transfer from neutrophils to macrophages is also possible. In infectious models, AM-derived EVs have been transferred to adjacent AMs and lung epithelial cells [28]. Additionally, macrophages phagocytose apoptotic neutrophils and monocytes [44, 45]. A lack of alveolar macrophages worsens influenza-related pneumonia and lung damage in mice, resulting in an increase of neutrophils and neutrophil extracellular traps [46]. Meanwhile, macrophages are responsible for removing excessive neutrophils, and the EVs released by macrophages increase the inflammatory response. As an alternative to removing macrophages, interfering with the excessive inflammation caused by EVs may represent a better strategy for treating sepsis-induced ALI. The interplay between protective and harmful innate and adaptive immune responses is complex, and hemostatic pathways play a crucial role in determining whether alveolar injury persists or resolves. Indeed, acute inflammatory responses to pathogens and their toxins cause acute lung injury (ALI) by releasing leukocyte proteases and initiating the production of reactive oxygen species (ROS), chemokines, and cytokines, as well as activating toll-like receptor (TLR) engagement and lipid mediators [47, 48].

## Conclusions

This study presents a novel method to predict the delivery of sEV-miRNAs to specific cells via correlation analysis of scRNA sequencing and sEV-miRNA target gene profiling. sEVs from patients with sepsis delivered miR-125a-5p to lung macrophages to inhibit Tnfaip3 and delivered miR-221-3p to neutrophils to inhibit Fos. sEVs from patients with sepsis also promoted ROS production in macrophages, as well as pro-inflammatory cytokine release from macrophages and neutrophils. EVs derived from LPS-stimulated BMDMs similarly induced oxidative stress in neutrophils. MM NPs containing miR-125a-5p inhibitor or miR-221-3p mimic reduced LPS-induced ALI in mice, while inhibiting TLR, CXCR2, or Tnfα reduced the lung oxidative stress caused by LPS-BMDM-EVs. Our findings propose novel treatment strategies and targets for intervening and reducing organ damage, especially lung damage, induced by EVs during sepsis.

## Experimental section/methods

### Ethics statement

Human blood samples were obtained from patients diagnosed with sepsis in the emergency department of Nanfang Hospital. Informed written consent was obtained from all patients. The protocol was approved by the Southern Medical University Ethics Committee (SMUC20181014). All animal experiments were performed following relevant national and international guidelines. The protocol was approved by the Southern Medical University Animal Care and Use Committee (2015044).

### Reagents and antibodies

LPS (E coli 0111: B4) was obtained from Sigma-Aldrich (St. Louis, MO, USA); BCA protein assay kits were acquired from Thermo Fisher Scientific (23,228; Waltham, MA, USA); protease cocktail inhibitors were acquired from Roche (Summerville, NJ, USA); and C188-9, Bay-11-7082, and CL-380,803 were purchased from Selleck (Houston, TX, USA). Clodronate liposomes were obtained from Vrije Universiteit Amsterdam (40337ES10; Netherlands); rabbit polyclonal CD63 (ab10895), CD9 (ab92726), Alix (ab117600), Tsg101 (ab30871), Ly6G (ab25377), and CXCR2 (ab14953) were sourced from Abcam (Cambridge, UK); and specific antibodies against CD81 (18250-1-AP), CD68 (28058-1-AP), Tnfaip3 (15104-1-AP), GAPDH (10494-1-AP), NF-κB (10745-1-AP), MPO (22225-1-AP), NOX4 (14374-1-AP), iNOS (18985-1-AP), and Tnfα (17590-1-AP) were obtained from Proteintech (Rosemont, IL, USA). Rabbit polyclonal p-NF-κB (3033 S) was obtained from Cell Signaling Technology (Boston, MA, USA), and primary antibody against Fos was obtained from Affinity (AF0132; Lancashire, UK). The drug library was purchased from Selleck.

### Cell lines and primary cell culture

Raw264.7 and HEK-293T cells were obtained from the Cell Bank of the Chinese Academy of Sciences (Shanghai, China). They were maintained in Dulbecco’s Modified Eagle’s Medium (DMEM) supplemented with 10% fetal bovine serum (FBS), 100 U/mL penicillin, 100 U/mL streptomycin, and 2 mM L-glutamine at 37 °C in a humidified incubator with 5% CO2. The cells were sub-cultured every 2 − 3 days. Human PMNs were isolated from a healthy control [49]. Primary bone marrow-derived neutrophil (BMDN) cells were isolated using a gradient centrifugation technique. Mouse femurs and tibias were used to prepare the BMDN cells. Bone marrow was flushed out using 5 mL of DMEM culture medium. The red cells in the bone marrow were lysed, and the remaining cells were resuspended with 65% Percoll (P1644; Sigma-Aldrich, St. Louis, MO, USA). This mixture was then overlaid onto 70% Percoll and was centrifuged at 750 × g for 30 min. BMDNs were collected from the interface and were centrifuged at 500 × g for 10 min. For the isolation of bone marrow-derived macrophages (BMDMs), mice were euthanized and sprayed with 75% ethanol. The femurs and tibias were then collected and utilized to prepare BMDMs following a standard protocol. Bone marrow was flushed out using 5 ml of BMDM culture medium consisting of DMEM supplemented with 10% FBS, 50 mg/mL penicillin/streptomycin, and 10 ng/mL of recombinant macrophage colony-stimulating factor (M-CSF) from Sigma-Aldrich. Red cells in bone marrow were lysed, and the remaining cells were suspended with BMDM culture medium at a concentration of 1 × 10^6^ cells/mL. The suspension was then seeded into culture dishes. On day 3, fresh BMDM medium was added, and it was changed once more on day 5. BMDMs were fully differentiated and ready for use by day 7. Next, BMDMs were seeded and incubated overnight to attach. The following morning, the cells were treated with LPS (100 ng/mL) for 24 h.

### EV isolation

BMDM-derived extracellular vesicles (BMDM-EVs) and serum extracellular vesicles (sEVs) were isolated and purified based on a previously described protocol [50]. In brief, BMDMs were treated with PBS or LPS (100 ng/mL) for 24 h, washed with PBS three times to remove LPS, and cultured in DMEM without FBS for an additional 24 h to collect the cell culture medium. The serum was then diluted with DMEM at a 1:5 ratio. A series of centrifugation steps were performed at 300 × g for 10 min, 2,000 × g for 20 min, and 10,000 × g for 30 min at 4 °C to obtain the medium. The supernatant was filtered through a 0.22-µm filter (Millipore, Bedford, MA, USA), collected, and then centrifuged for 70 min at 4 °C at 100,000 × g using a Beckman Coulter Optima TM L-80XP (Brea, CA, USA) in order to pellet EVs. The EVs were subsequently washed with sterile PBS, centrifuged again for 70 min at 4 °C at 100,000 × g, and then carefully reconstituted in sterile PBS or lysed in RIPA buffer.

### Transmission electron microscopy

Purified EVs and synthetic vesicles were fixed in a solution containing 2.0% glutaraldehyde and 4% sucrose in a 0.05 M phosphate buffer (pH 7.4) for a duration of 2 h. Afterward, they were treated with 1% osmium tetroxide for 1 h at room temperature, followed by block-staining in 0.5% aqueous uranyl acetate, dehydration via increasing concentrations of ethanol, and embedding in Epon 812. Ultrathin sections were prepared with an RMC MT6000-XL ultramicrotome (Sorvall, Waltham, MA, USA) and imaged using a JEM-2100 F transmission electron microscope (JEOL Ltd., Tokyo, Japan).

### Nanoparticle tracking analysis

The particle size and concentration distribution of the isolated EVs were measured via NTA (Multiple-Laser ZetaView f-NTA Nanoparticle Tracking Analyzer) according to the manufacturer’s instructions. Briefly, the EV samples were diluted to 1:500 with filtered sterile PBS, and each sample was analyzed for 60 s in triplicate using Nanosight automatic analysis settings.

### EV labeling and immunofluorescent staining

Serum EVs were labeled with PKH67 dye obtained from Sigma-Aldrich (St. Louis, MO, USA). First, PBS-diluted EVs were added to 1 mL of diluent C. In parallel, 4 µL of PKH67 dye was added and incubated with the EV solution for 4 min. Following this, BSA (2 mL 0.5%) was incorporated, and labeled EVs were washed at 100,000 × g for 1 h. For in vivo uptake experiments, PKH67-labeled EVs (100 µg) were injected into the tail veins of mice. Liver and lung tissues were snap-frozen in liquid nitrogen and embedded in tissue-Tek OCT compound for further analysis. Tissue samples were fixed and cut into 5-µm sections using a cryostat. The sections were then washed in PBS before being stained with DAPI for nuclear imaging. The imaging procedure was conducted using a Zeiss Axio Imager 2 microscope (Jena, Germany) and processed with Zeiss ZEN software (Jena, Germany).

### Single-cell RNA sequencing

Liver and lung tissues were resuscitated and washed using PBS containing 0.1% BSA, followed by cell washing with 1nmL of stain buffer (also containing 0.1% BSA) and subsequent cell counting. Cell viability of all samples exceeded 80%. Subsequently, cells were labeled, counted, and multiplexed using the BD Human Single-Cell Multiplexing Kit (BD Biosciences, San Jose, CA, USA). Single-cell capture and cDNA synthesis were conducted utilizing the BD Rhapsody Single-Cell Analysis System (BD Biosciences, Franklin Lakes, NJ, USA) following the manufacturer’s guidelines. An Illumina NextSeq (San Diego, CA, USA) was used to sequence the libraries over multiple runs.

### Unsupervised clustering of cells and t-distributed stochastic neighbor embedding-visualization

Unsupervised cell clustering was conducted using the BD Rhapsody Single-Cell (SC) Analysis System (BD Biosciences) and SC-sequencing data. Genes expressed in less than two cells were excluded. Cells with more than 200 genes and less than 10% mitochondrial genes were subjected to further processing. Seurat arithmetic was employed to determine the variation coefficient of genes. Dimensionality reduction of all data was performed using principal component analysis (PCA) based on the top 1,500 DEGs. A k-nearest neighbor graph was constructed using Euclidean distances in the space of the first 10 significant principal components. The cells were then clustered using the Louvain Modularity optimization algorithm, and the resulting clusters were visualized with the tSNE project. Cells with high expression of hemoglobin were excluded from the analyses.

### Identification of marker genes and cell-type annotation

Differential expression of each cluster was calculated using the bimod test as implemented in the BD Rhapsody Single-Cell Analysis System (BD Biosciences, Franklin Lakes, NJ, USA). Genes with a log2 mean differential expression of at least 0.585 and a P-value < 0.05 were considered marker genes. Canonical markers that are associated with known cell types were employed to annotate the cell clusters.

### Differential expression analysis, clustering, and heatmaps

Differential expression analysis was performed on the reconstructed expression using SeqGeq (BD Biosciences). We selected DEGs with a false discovery rate of < 5% and a fold change value of ≥ 1.3 between the sepsis sEV-treated and healthy control sEV-treated groups. Gene sets were clustered using hierarchical clustering with complete linkage and were visualized using heatmaps. Gene set enrichment analyses were performed on these gene sets and clusters using Fisher’s test.

### Serum EV-miRNA sequencing

sEV RNA was extracted utilizing a HiPure Liquid miRNA Kit or HiPure Serum/Plasma miRNA Kit produced by Megan in China. The amount and quality of sEV RNA were evaluated individually using the Qubit 2.0 (Life Technologies, Carlsbad, CA, USA) and Agilent 2200 TapeStation (Agilent Technologies, Palo Alto, CA, USA). For the preparation of small RNA libraries, 50 ng of sEV RNA from each sample was utilized with a NEBNext Multiplex Small RNA Library Preparation Set for Illumina (NEB, Ipswich, MA, USA) per the manufacturer’s guidelines. The sequencing of the libraries was performed using a HiSeq 2500 (Illumina, San Diego, California, USA) with single-end 50-bp primers from Ribobio Co., Ltd. (Guangzhou, China). The raw data underwent refinement by excluding reads that contained an adapter, poly “N,” low quality reads, and those < 17 nt by FASTQC. Clean reads were aligned to a reference genome using Burrows-Wheeler Aligner (BWA). Known mature miRNA were identified based on miRBase21 (www.miRBase.org) using miRDeep2, and novel miRNA were predicted. MiRNA expression was determined by RPM values [RPM = (number of reads mapped to miRNA/number of clean data reads) × 10^6^]. The expression levels were normalized by RPM. The edgeR algorithm was used to calculate the differential expression between two sample sets based on the criteria of |log2 (fold change)| ≥ 1 and P-value < 0.05. TargetScan, miRDB, miRTarBase, and miRWalk were employed to predict the target genes of selected miRNA.

### Transcriptomic sequencing

Total RNA was extracted using a TRIzol reagent kit acquired from Invitrogen (USA) and assessed for quality on an Agilent 2100 Bioanalyzer from Agilent Technologies (USA). Following RNA extraction, eukaryotic mRNA was enriched using Oligo (dT) beads. Prokaryotic mRNA was enriched by removing rRNA with a Ribo-ZeroTM Magnetic Kit from Epicentre (Madison, WI, USA). The enriched mRNA was fragmented into short segments using a fragmentation buffer and then reverse transcribed into cDNA with random primers. Second-strand cDNA was synthesized using DNA polymerase I, RNase H, dNTP, and buffer. The resulting cDNA fragments were purified with a QiaQuick PCR extraction kit from Qiagen (Venlo, Netherlands), end-repaired, poly(A) added, and ligated to Illumina sequencing adapters. The ligation products underwent size selection through agarose gel electrophoresis, were PCR-amplified, and sequenced utilizing the Illumina HiSeq 2500 system from Gene Denovo Biotechnology Co. (Guangzhou, China).

DESeq2 software and edgeR were employed for differential expression analysis of the two RNA groups. Only genes/transcripts displaying a false discovery rate (FDR) of < 0.05 and an absolute fold change value of ≥ 2 were deemed differentially expressed. Principal component analysis (PCA) was conducted, and all differentially expressed genes (DEGs) were mapped to Gene Ontology (GO) terms in the Gene Ontology database. Gene numbers were calculated for each term, and significantly enriched GO terms were identified by comparing them with the genome background using the hypergeometric test. Kyoto Encyclopedia of Genes and Genomes (KEGG) pathway enrichment analysis detected significantly enriched metabolic or signal transduction pathways of DEGs compared to the whole genome background. Protein-protein interactions were identified through the use of STRING v10. The resulting network was presented using Cytoscape (v3.7.1) software to highlight core and hub gene interactions.

### Proteomic analysis

The sEVs were isolated from the sample and SDT buffer was subsequently added. The lysate was boiled for 15 min and then centrifuged at 14,000 × *g* for 40 min. The resulting supernatant was quantified using the BCA Protein Assay Kit (P0012, Beyotime, Shanghai, China). Proteins (20 µg) were mixed with 6X loading buffer and boiled for 5 min before being separated on a 12.5% SDS-PAGE gel. Additionally, proteins (200 µg) from each sample were incorporated into 30 µL of SDT buffer (4% SDS, 100 mM DTT, 150 mM Tris-HCl pH 8.0) prior to analysis. The peptide mixture (100 µg) from each sample underwent labeling with TMT reagent per the manufacturer’s instructions (Thermo Fisher Scientific, MA, USA). Subsequently, using a Q Exactive HF-X mass spectrometer (Thermo Fisher Scientific, MA, USA) coupled with Easy-nLC (Thermo Fisher Scientific, MA, USA), NanoLC-MS/MS analysis was conducted for each fraction. MS/MS raw files were processed using version 2.6 of the MASCOT engine developed by Matrix Science (London, UK), which was embedded into Proteome Discoverer 2.2. The search was performed against the Uniprot_HomoSapiens_20367_20200226 database. To enforce a peptide and protein false discovery rate of 1%, a reverse database search strategy was implemented. Proteins were flagged as differentially expressed if they displayed a fold change value greater than 1.2 and a P-value less than 0.05, determined by a Student’s t-test. The GO term selected was the one associated with the sequence having the highest Bit-Score determined by Blast2GO. Afterward, the annotation of GO terms to proteins was carried out using the Blast2GO Command Line. Following the elementary annotation, InterProScan was utilized to search the EBI database by motif. The functional information of motif to proteins was then added to enhance the annotation. ANNEX was employed to further improve the annotation and establish connections between GO terms. The Fisher exact test was utilized to enrich GO terms by comparing the number of proteins correlated to GO terms that were differentially expressed and the total proteins. The KEGG database was used for pathway analysis. The Fisher exact test was also employed to identify significantly enriched pathways by comparing the number of differentially expressed proteins and the total proteins correlated to each pathway. The target protein’s gene symbol was utilized to locate interactions, both direct and indirect, in the STRING-db database. Subsequently, an interaction network was generated and analyzed via Cytoscape software (v3.7.1).

### Preparation and characterization of MM-vesicles

MM-vesicles were prepared according to previous reports with some minor alterations [51]. In short, RAW 264.7 cells were cultured before being detached with 2 mM EDTA PBS solution. The cells were then washed three times with PBS and resuspended in a hypotonic lysing buffer containing 2 mM MgCl_2_, 10 mM KCl, 20 mM Tris-HCl, and 1 EDTA-free mini protease inhibitor tablet per 10 mL solution. Subsequently, a Dounce homogenizer was used to disrupt them. The entire solution was passaged 20 times before being spun down at 3,200 × *g* for 5 min. The supernatants were retained and centrifuged at 20,000 × *g* for 30 min, after which the pellet was discarded. The pellets containing the cell membranes were washed once in a solution of 1 mM EDTA and 10 mM Tris-HCl, before being collected as purified MM. Later on, MM-vesicles were produced by physically extruding the pellets through 1,000-nm and 400-nm microporous membranes for several passes, using an Avanti mini extruder (Avanti Polar Lipids, Alabaster, AL, USA). The dynamic light scattering technique (DLS; Nano-Zen 3600, Malvern Instruments, Great Malvern, UK) was used to measure the products’ diameter.

### Preparation and characterization of miR-125a-5p inhibitor@MM nanoparticles and miR-221-3p mimic@MM NPs

PBS (1 mL) containing 50 nmol miR-125a-5p inhibitors (or miR-221-3p mimics) was mixed with MM-vesicles and extruded 14 times through a 200-nm membrane. The resulting miR-125a-5p inhibitor@MM nanoparticles (NPs) and miR-221-3p mimic@MM NPs remained in PBS at 4 °C for further animal experiments. The morphology of miR-125a-5p inhibitor@MM NPs and miR-221-3p mimic@MM NPs were characterized using TEM. The miR-125a-5p inhibitors or miR-221-3p mimics were labeled with Alexa Flour 488 according to the manufacturer’s instructions (Invitrogen, USA), and the loading efficiency of Alexa Flour 488- miR-125a-5p inhibitors (or miR-221-3p mimics) in MM NPs were determined by detecting the fluorescence intensity of MM NPs.

### Imaging of in vivo MM NPs

For labeling of MM nanoparticles, 5 µL of DiR (Life Technologies, California, USA) were mixed at a concentration of 200 µg/mL in ethanol with 200 µg of MM NPs in 100 µL of PBS for 15 min in the dark. The mixture was then centrifuged at 13,000 × g for 30 min to remove ethanol and unincorporated DiR. Images were captured via Spectral Instruments Imaging (Tucson, Arizona) using a 748 nm excitation wavelength and a 780 nm filter to detect the fluorescence signals of DiR over a range of time intervals: 0 min, 1 min, 5 min, 10 min, 20 min, 30 min, 1 h, 3 h, 12 h, 24 h, and 48 h.

### Induction of ALI and experimental designs

C57BL/6 mice, aged 8–12 weeks, were acquired from the Experimental Animal Center of Southern Medical University in Guangzhou, China. The mice were housed in a pathogen-free environment with 12-hour light–dark cycles and were provided with ad libitum access to food and water. To induce acute lung injury (ALI), the mice were administered a single intraperitoneal dose of 15 mg/kg of LPS.

To evaluate the impact of MM NPs on LPS-induced acute lung injury (ALI), we randomly assigned all animals to five groups: control, LPS, LPS + miR-125a-5p inhibitor@MM NP, LPS + miR-221-3p mimic@MM NP, and LPS + miR-125a-5p inhibitor + miR-221-3p mimic@MM NP groups. The mice received aerosolized MM NPs via intratracheal liquid injection (Shanghai Yuyan Instruments Co., Ltd., Shanghai, China) for 48 h before being intraperitoneally injected with LPS (15 mg/kg). In contrast, sham mice were given the same volume of saline. After 4 h, six animals per group were sacrificed to gather lung tissue, while 10 animals per group were reserved for survival analysis. The survival analysis lasted for 7 days, with survival monitored every 6 h. The endpoint of the survival experiment was reached 7 days after LPS administration, upon which the survival rate was analyzed. All surviving mice were euthanized through cervical dislocation using an anesthesia mixture of ketamine and xylazine. To determine the percentage of MM NPs targeted to macrophages in the lung after tracheal administration. The mice received aerosolized FITC-labeled MM NPs via intratracheal administration, and the percentage of macrophages (PE-labeled F4/80 positive cells) that absorbed FITC-MM NPs was detected using flow cytometry. CL-380,803 (100 mg/kg i.p.), leonurine hydrochloride (100 mg/kg i.p.), or SB225002 (1 mg/kg i.p.) were administered to the mice 30 min before LPS (15 mg/kg) injection to evaluate the inhibitors’ effect in sepsis. Mice were anesthetized at 8 h after LPS injection. Subsequently, the lungs were harvested to conduct histological and immunohistochemical analysis. RNA and protein were directly extracted as soon as possible.

### Luciferase reporter assay

HEK-293T cells were co-transfected with 0.5 mg of luciferase reporters for wild-type or mutant-type 3forct, which included the *Renilla* firefly luciferase gene and miR-125a-5p-NC/miR-221-3p-NC or miR-125a-5p mimic/miR-221-3p mimic. *Renilla* luciferase reporters were used as an internal control.

### Protein isolation and Western blot

To isolate total protein, lysing of cultured cells or EVs was carried out with RIPA buffer (P0013B, Beyotime, Jiangsu, China). Subsequently, the measurement of protein concentration was carried out via the use of an Enhanced BCA Protein Assay Kit (Beyotime, Jiangsu, China). Next, aliquots of total protein extracts (20 µg) were loaded and separated by a 10% SDS-PAGE gel. This was followed by a transfer onto a PVDF membrane (Millipore) for western blotting. After being blocked for one hour with blocking buffer, the membranes were incubated with polyclonal antibodies targeting CD63, CD9, CD81, Alix, Tsg101, CXCR2, GAPDH, MPO, NOX4, iNOS, Tnf-α, and p-NF-κB. Afterward, the membranes were washed three times with PBS and incubated with appropriate secondary antibodies (LI-COR Bioscience, Lincoln, NE, USA). Finally, protein bands were detected using the Odyssey system (LI-COR Bioscience).

### Quantitative reverse transcription polymerase chain reaction (RT-qPCR)

Total RNA from cultured cells or mouse lungs was extracted using TRIzol reagent acquired from Invitrogen (USA) following the manufacturer’s instructions. Subsequently, the ReverTra Ace qPCR RT Kit (FSQ-101, Toyobo) was used to synthesize cDNA, also following Invitrogen’s guidelines. Primers were manufactured by BGI (Beijing, China). RT-qPCR was performed using SYBR qPCR mix (QPK-201, ToYoBo) in Roche 480 system, with 18s serving as the internal control. The expression levels of miRNA were quantified by two-step RT-qPCR. Total mRNA was isolated using TRIzol reagent, and the cDNA was synthesized using the miDETECT A Track miRNA qRT-PCR Starter Kit in accordance with the manufacturer’s instructions (Ribobio). The miRNA primers (hsa-miR-125a-5p, hsa-miR-221-3p) were generated by Ribobio and U6 was used as an internal control. The primers used to generate the specific products were designed as presented in Supplement Table 1. All reactions were carried out in triplicate. The expression levels are relative to the corresponding control samples taken at the same time point. The selected gene’s threshold cycle (Ct) values were standardized against 18s (or U6) values of the same sample. Using the comparative Ct method (△△Ct), the fold changes between the samples were determined. The results represent the mean ± SD expression of each gene, calculated from at least three determinations per gene.

### Histopathology and immunohistochemistry

Lung tissue embedded in paraffin was cut into 5-µm-thick slices and stained with hematoxylin and eosin (H&E). Histological features were assessed using a 0–3 scale (0 representing normal, 2 indicating moderate, and 3 pointing towards severe). The overall score of lung damage was computed based on the sum of the scores of alveolar edema and hemorrhage, leukocytes infestation, and thickness of alveolar walls and epithelium. For immunohistochemical staining, the liver and lung sections were deparaffinized, rehydrated, and treated with a 3% H2O2 solution for 10 min. Antigen retrieval was then performed for 15 min in a citrate buffer. Nonspecific protein was blocked with 10% goat serum for 30 min. The lung slides were incubated with diluted primary antibodies against CD68, Ly6G, Tnfaip3, Fos, MPO, NOX4, and iNOS according to the manufacturer’s protocols. Liver tissue samples were incubated with primary antibodies against Ly6G in accordance with the manufacturer’s instructions. A biotin-conjugated anti-rabbit IgG antibody was used to detect the primary antibody, followed by incubation with streptavidin-biotin. The complex was visually examined using DAB reagent under a microscope (Zeiss, Germany) at 200-fold magnification, and samples were subsequently analyzed and photographed.

### Enzyme-linked immunosorbent assay (ELISA)

LPS quantification was executed through quantitative ELISA (2 M-KMLJM219755m, Camilo). An ELISA-dedicated instrument was employed to measure the optical densities at 450 nm, and LPS concentrations were calculated using a standard curve. The quantification was carried out in duplicate, and arithmetic averages were calculated.

### Analysis of total ROS levels

The ROS levels were assessed with DCFDA (S0033; Beyotime). Thp-1 or primary murine BMDNs were incubated with 10 µM DCFDA at 37 °C for 30 min. The cells were then washed with PBS and analyzed using a flow cytometer (BD FACSCanto II; BD Biosciences).

### Statistical analysis

Data are expressed as the mean ± SD. Statistical comparisons were performed using one-way ANOVA or two-tailed t test. Survival rates were analyzed with the Kaplan–Meier test. A P-value < 0.05 was considered statistically significant. All statistical analyses were performed with GraphPad Prism 6 software.

### Electronic supplementary material

Below is the link to the electronic supplementary material.

Supplementary Material 1

## Data Availability

All data generated or used during the study are available from the corresponding author upon reasonable request.

## Abbreviations

ABs: Apoptotic bodies
ALI: Acute lung injury
AMs: Alveolar macrophages
ARDS: Acute respiratory distress syndrome
BALF: Bronchoalveolar lavage fluid
BMDMs: Bone marrow-derived macrophages
BMDNs: Bone marrow-derived neutrophils
BSA: Bovine serum albumin
DAMPs: Damage associated molecular patterns
DEGs: Differentially expressed genes
DMEM: Dulbecco’s Modified Eagle’s Medium
E. coli: Escherichia coli
ELISA: Enzyme-linked immunosorbent assay
EVs: Extracellular vesicles
FBS: Fetal bovine serum
FITC: Fluorescein isothiocyanate
GO: Gene Ontology
HC: Healthy control
H&E: Hematoxylin-eosin
IHC: Immunohistochemistry
iNOS: Inducible nitric oxide synthase
KEGG: Kyoto Encyclopedia of Genes and Genomes
LPS: Lipopolysaccharide
lncRNA: Long non-coding RNA
M-CSF: Macrophage colony-stimulating factor
MM: Macrophage membrane
MM NPs: Macrophage membrane nanoparticles
MSN: Mesenchymal stem cells
MVs: Microvesicles
NTA: Nanoparticle tracking analysis
PAMPs: Pathogen-associated molecular patterns
PCA: Principal component analysis
PMN: Polymorphonuclear neutrophil
ROS: Reactive oxygen species
RT-qPCR: Quantitative reverse transcription polymerase chain reaction
scRNA: Single-cell RNA
sEV: Serum EV
SOFA: Sequential organ failure assessment
TEM: Transmission electron microscopy
TLR: Toll-like receptor
tSNE: t-distributed stochastic neighbor embedding
