# Supplementary material for: Macrophage biomimetic nanoparticle-targeted functional extracellular vesicle micro-RNAs revealed via multiomics analysis alleviate sepsis-induced acute lung injury
Source: J Nanobiotechnology. 2024 Jun 23;22:362. doi: 10.1186/s12951-024-02597-z (PMC11194988; doi:10.1186/s12951-024-02597-z)
Supplement: Supplementary file 1 — Supplementary Material 1 [file 12951_2024_2597_MOESM1_ESM.docx]

**Supporting Information**.

**Table S1.** List of Primers used for RT-qPCR.

| **Gene** | **Forward Primer (5`-3`)** | **Reverse Primer (5`-3`)** |
| --- | --- | --- |
| Mou-Tnf-a | CCACCACGCTCTTCTGTCTAC | AGGGTCTGGGCCATAGAACT |
| Mou- IL-6 | TGATGCACTTGCAGAAAACA | ACCAGAGGAAATTTTCAATAGGC |
| Mou- IL-1b | GGTCAAAGGTTTGGAAGCAG | TGTGAAATGCCACCTTTTGA |
| Mou-Cxcl1 | ACCCAAACCGAAGTCATAGC | TCTCCGTTACTTGGGGACAC |
| Mou-Cxcl2 | CGGTCAAAAAGTTTGCCTTG | TCCAGGTCAGTTAGCCTTGC |
| Mou-Cxcl10 | CTCATCCTGCTGGGTCTGAG | CCTATGGCCCTCATTCTCAC |
| Mou-CCL2 | CCTGCTGTTCACAGTTGCC | ATTGGGATCATCTTGCTGGT |
| Mou-Ccl3 | ACCATGACACTCTGCAACCA | GTGGAATCTTCCGGCTGTAG |
| Mou-Ccl5 | GTGCCCACGTCAAGGAGTAT | CCACTTCTTCTCTGGGTTGG |
| Mou-TNFAIP3 | CATAGAGACATGCCTCGAACTA | TAACCATTACACTTGGCATTGC |
| Hum-TNFAIP3 | CACCAGCGTTCCAAGTCAGATCC | TTGCTCGTCCCCGTCCTGTC |
| Mou-Fos | TCTCTAGTGCCAACTTTATCCC | GAGATAGCTGCTCTACTTTGCC |
| Hum-Fos | CTTCCCAGAAGAGATGTCTGTG | TGGGAACAGGAAGTCATCAAAG |
| Mou-IRF7 | GCGTACCCTGGAAGCATTTC | GCGTACCCTGGAAGCATTTC |
| Mou-Oasl1 | TGCTCAAGGTACTCAAGGTAGG | TGGGTACTCTGTTAGTCACACTC |
| Mou-Ifit1 | GTCCGGTTAAATCCAGAAGATCC | GCTTTGTCTACGCGATGTTTCC |
| Mou-18s | CGATCCGAGGGCCTCACTA | AGTCCCTGCCCTTTGTACACA |
| Hum-18s | AGGAATTCCCAGTAAGTGCG | GCCTCACTAAACCATCCAA |


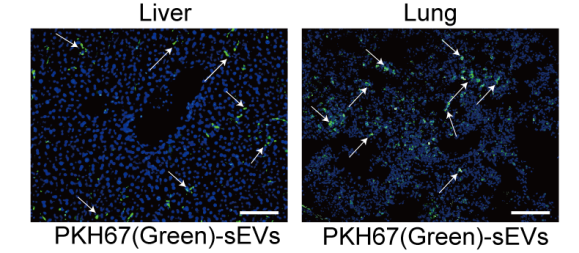


**Figure S1** Uptake of PKH67-labeled sEVs from the serum by mouse liver and lung tissues.


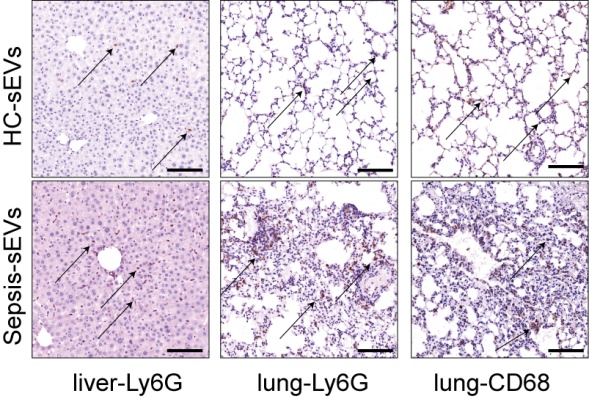


**Figure S2.** Ly6G and CD68 staining in mouse (n = 6 per group) liver and lung tissues after exposure to healthy or sepsis sEVs.


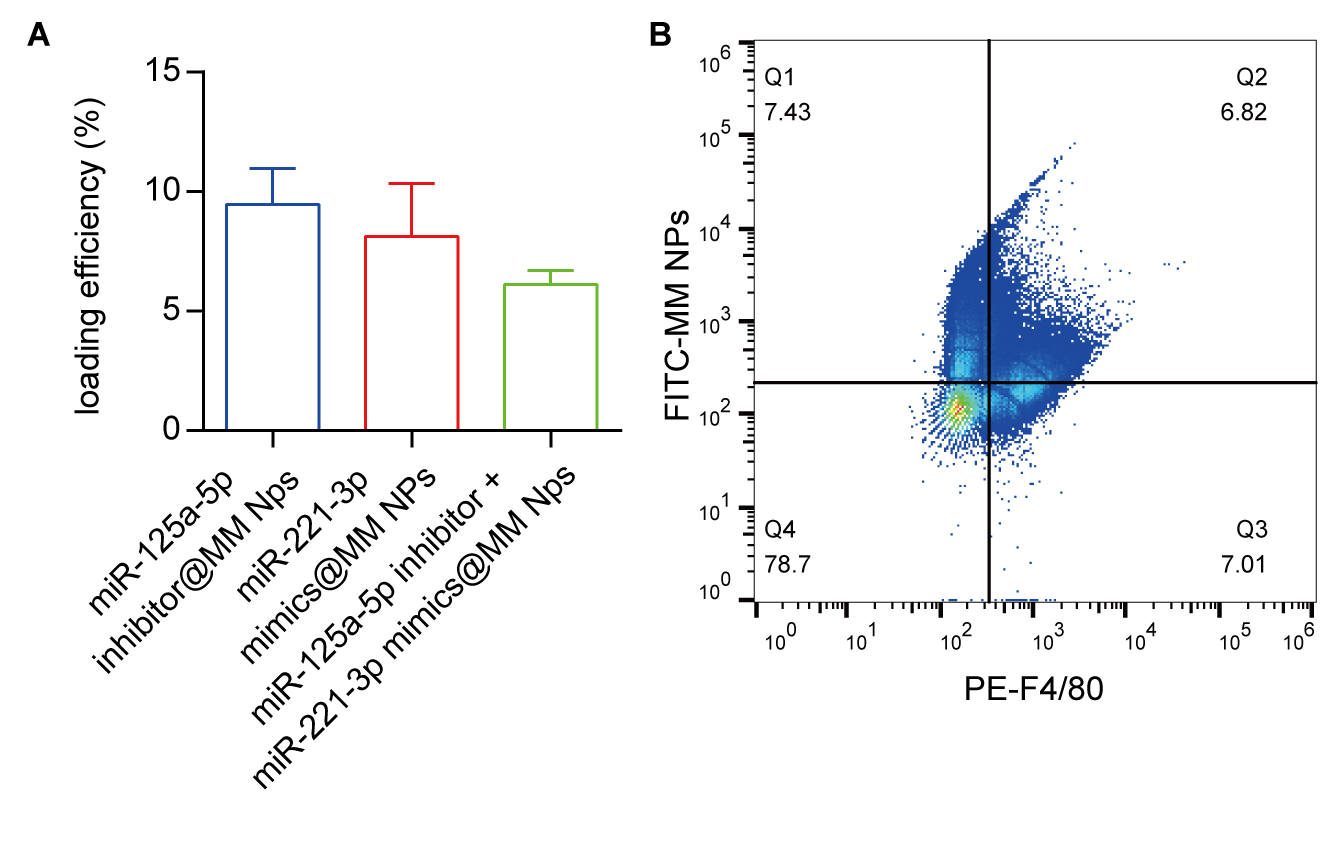


**Figure S3.** The loading efficiency of miR-125a-5p inhibitors (or miR-221-3p mimics) in MM NPs and MM NPs targeted to macrophages in the lung. (A) The loading efficiency of miR-125a-5p inhibitors (or miR-221-3p mimics) detected by the fluorescence intensity of FITC-labeled miR-125a-5p inhibitors (or miR-221-3p mimics) in MM NPs. (B) The percentage of macrophage (F4/80 positive cells) that absorbed FITC positive MM NPs by flow cytometry.


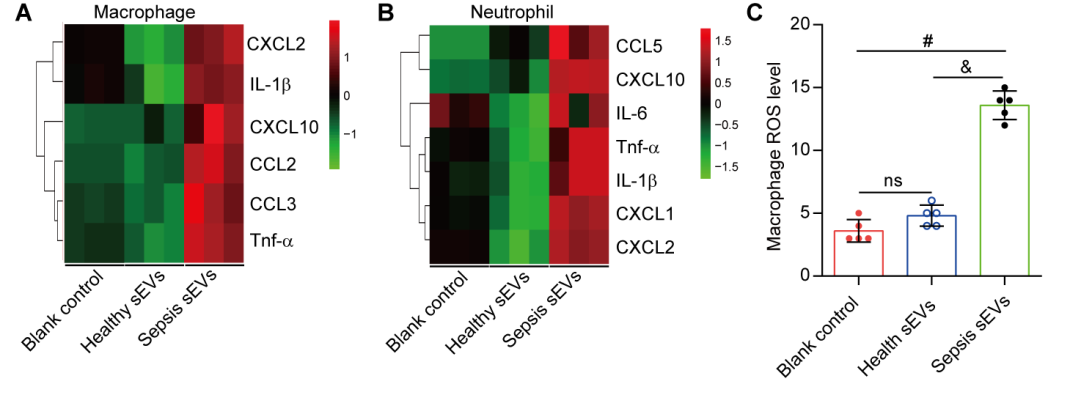


**Figure S4.** Serum EVs from patients with sepsis promote ROS release from macrophages and pro-inflammatory cytokine release from macrophages and neutrophils. (A) Levels of Tnfα, IL-1β, CXCL2, CXCL10, CCL2, and CCL3 increased significantly in response to sepsis sEVs compared with healthy sEVs. (B) Levels of Tnfα, IL-6, IL-1β, CXCL1, CXCL2, CCL5, and CXCL10 increased significantly in response to sepsis sEVs compared with those from the control group and healthy individuals. (C) The ROS levels increased significantly in response to sepsis sEVs compared with healthy sEVs. ^&^P < 0.05 versus healthy sEVs.


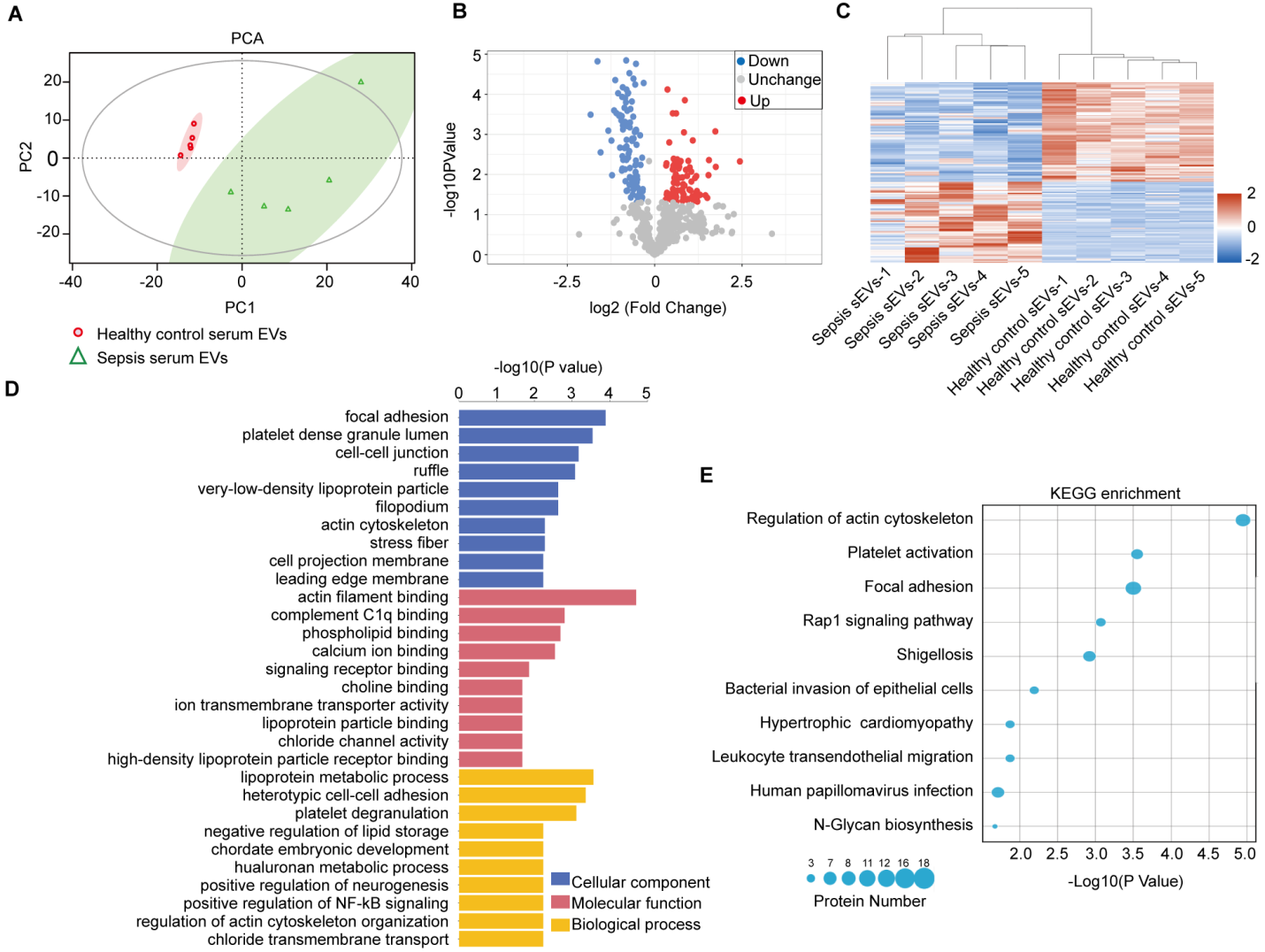


**Figure S5.** Mass spectrometry of serum EVs (sEVs) from healthy individuals and patients with sepsis. (A) PCA of the mass spectra of sEVs from healthy individuals and patients with sepsis. (B) Volcano plot of the mass spectra of sEVs from healthy individuals and patients with sepsis. (C–E) Heatmap, GO analysis, and KEGG analysis of the mass spectra of sEVs from healthy individuals and patients with sepsis.


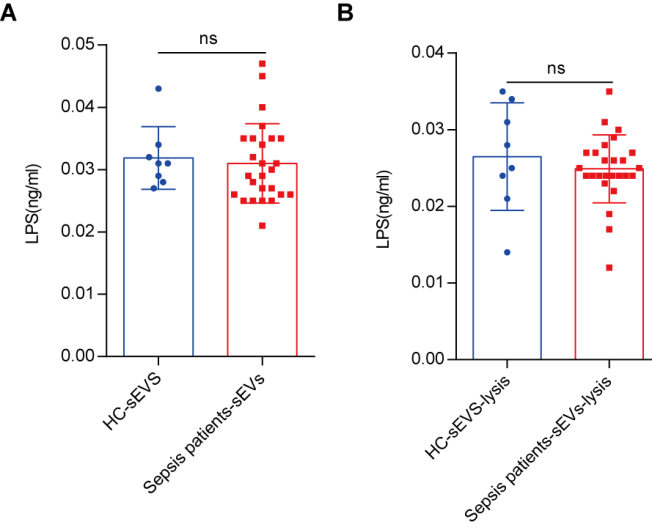


**Figure S6.** Levels of LPS in HC-sEVs, sepsis sEVs, and lysed EVs.


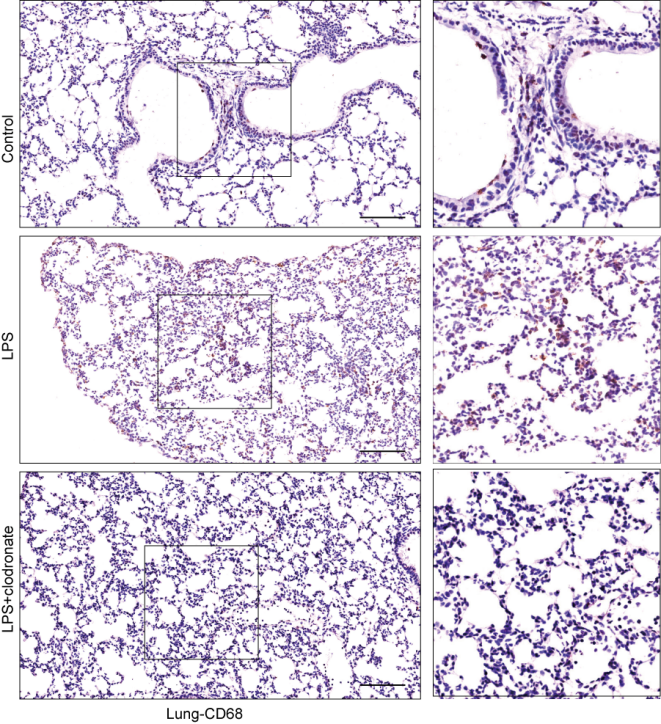


**Figure S7.** Analysis of CD68 expression levels in the lung (n = 6 per group) using IHC.


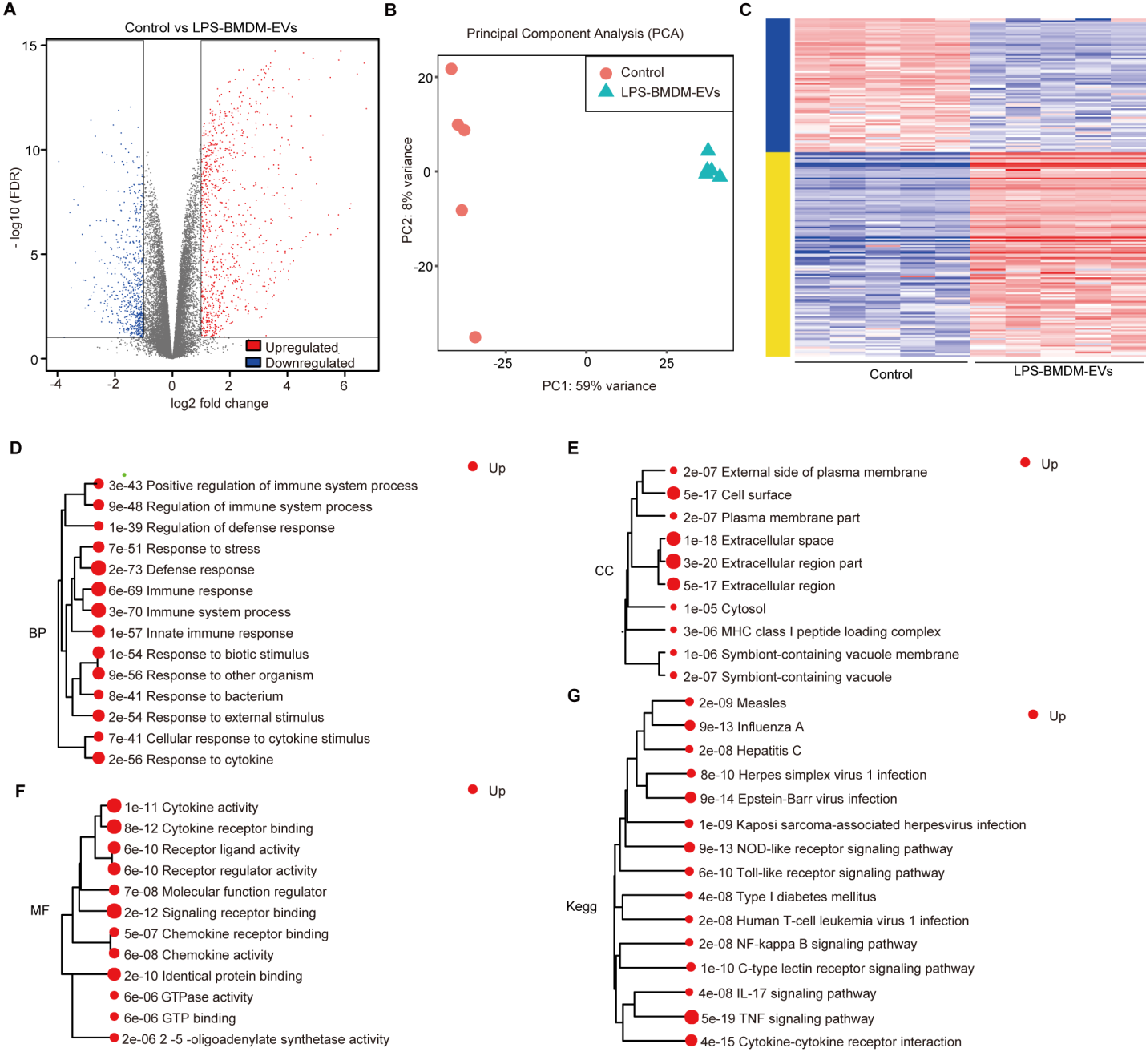


**Figure S8.** Transcriptome profiles of neutrophils exposed to EVs derived from LPS-BMDM. (A) Volcano map of the genetic changes in neutrophils after treatment with LPS-BMDM-EVs relative to the control treatment (n = 5 per group). (B) PCA of the control and LPS-BMDM-EV treatment groups (n = 5 per group). (C) Heatmap of neutrophil DEGs in the control and LPS-BMDM-EV treatment groups (n = 5 per group). (D–G) Biological process (BP), cellular component (CC), molecular function (MF), and KEGG analyses of neutrophils in the control and LPS-BMDM-EV groups. PCA: principal component analysis; BMDM: bone marrow-derived macrophage; and DEG: differentially expressed gene.


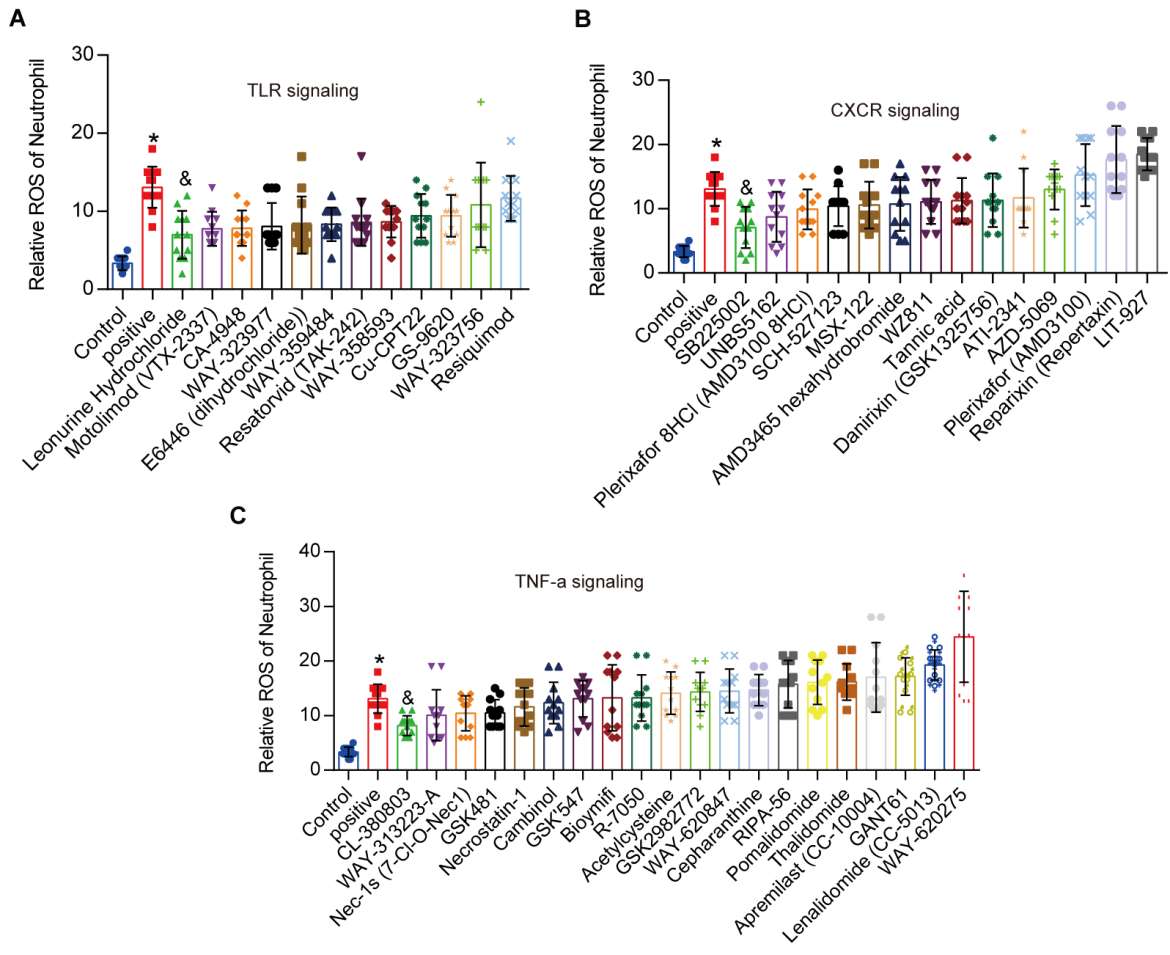


**Figure S9.** ROS level in neutrophils. (A–C) Effect of TLR, CXCR, and Tnfα signaling pathway inhibitors on the ROS levels (mean ± SD, n = 8 per group) of neutrophils induced by LPS-BMDM-EVs. *: P < 0.05 versus control; &: P < 0.05 versus positive group.
